# Advances in Ablation or Oxidation Mechanisms and Behaviors of Carbon Fiber-Reinforced Si-Based Composites

**DOI:** 10.3390/molecules28166022

**Published:** 2023-08-11

**Authors:** Hongmei Zuo, Fangtao Ruan, Hongjie Wang, He Wang, Xu Wang, Yufan Huang, Rui Wang, Lihua Zou, Zhenzhen Xu, Diansen Li

**Affiliations:** 1 School of Textile and Garment, Anhui Polytechnic University, Wuhu 241000, China; zuohongmei@ahpu.edu.cn (H.Z.); ruanfangtao@ahpu.edu.cn (F.R.); wanghongjie@ahpu.edu.cn (H.W.); wanghe@ahpu.edu.cn (H.W.); wangxu_ahpu@icloud.com (X.W.); huangyu0511@126.com (Y.H.); x1261560441@126.com (R.W.); 2 Chery New Energy Automobile Co., Ltd., Wuhu 241003, China; 3 Anhui Key Laboratory of New Energy Automobile Lightweight Technology, Wuhu 241003, China; 4 Beijing Key Laboratory of Bio-Inspired Energy Materials and Devices, School of Chemistry, Beihang University, Beijing 100191, China; lidiansen@buaa.edu.cn; 5 Beijing Advanced Innovation Center for Biomedical Engineering, Beihang University, Beijing 100191, China

**Keywords:** carbon fiber-reinforced composites, oxidation, ablation, Si-based ceramics, mechanisms

## Abstract

Composites with excellent thermomechanical and thermochemical properties are urgently needed in the aerospace field, especially for structural applications under high-temperature conditions. Carbon fiber-reinforced Si-based composites are considered the most promising potential high-temperature materials due to their excellent oxidation resistance and ablative behaviors, good structural designability, and excellent mechanical properties. The reinforcement of the relevant composites mainly involves carbon fiber, which possesses good mechanical and temperature resistance abilities. In this paper, the ablation behaviors and mechanisms of related composites are reviewed. For carbon fiber-reinforced pure Si-based composites (C/SiM composites), the anti-ablation mechanism is mainly attributed to the continuous glassy SiO_2_, which inhibits the damage of the substrate. For C/SiM composite doping with refractory metal compounds, the oxides of Si and refractory metal together protect the main substrate from ablation and oxidation. Moreover, in addition to thermochemical damage, thermophysical and thermomechanical behavior severely destroy the surface coating of the substrate.

## 1. Introduction

In the field of aerospace, advanced thermal protection systems related to aerospace flight and rocket propulsion require some special materials, which have not only excellent thermal shock resistance, light weight, and high strength, but also excellent ablative resistance [1,2,3]. In practical application, ablation and active oxidation are severe problems, which must be avoided [4]. The mechanical properties of these materials have also been widely researched [5,6]. Therefore, it is urgent to develop ultrahigh-temperature materials with a high melting point to meet the application requirements in extreme-temperature environments above 1600 °C, especially when they are used as the leading edges and nose cones of hypersonic aircraft [7,8,9].

Fiber-reinforced composites with carbon fiber as the reinforcement material possess not only excellent mechanical properties, but also good thermal shock and ablative resistance [10]. They can be applied in extreme environments such as ultrahigh-temperature structures [11,12,13]. However, as the reinforcing material, carbon fiber is susceptible to oxidization above 450 °C in aerobic environments, which limits its application [14,15,16]. At present, there are several methods to improve the ablative and oxidation resistance of fiber-reinforced composites. The first approach is the use of a ceramic fiber or other anti-ablative reinforcements, such as SiC fiber [17], which have better ablative properties, especially in oxygenated environments. When ceramic fiber-reinforced matrix composites are coated with environmental barrier coatings, they can be used in jet engines, as extensively and intensively reported in recent reviews by Fang et al. [18] and Tejero-Martin et al. [19]. The second approach is the optimization of the fiber structure (using 2.5D or 3D textiles). The third approach is an improvement of the interfacial bonding between the fiber and matrix, where pyrolytic carbon (PyC) is generally added between the fiber and matrix. The fourth approach is the addition of an ultrahigh-temperature ceramic into matrix. The fifth approach is coating the fibers or textile structure with ablative ceramics to suppress oxygen diffusion. By implementing the above measures, when ceramics with excellent ablative resistance are used as the matrix, and fibers with outstanding mechanical properties are used as reinforcement material, the composites can be applied to hypersonic vehicles or other high-temperature aerobic environments.

**Figure 1:**
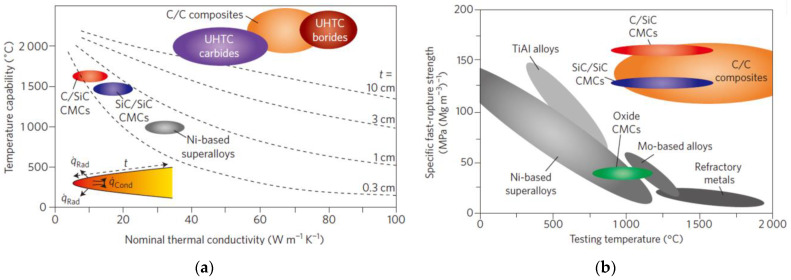
The optimal performance temperature and outstanding mechanical properties of carbon fiber-reinforced Si-based ceramic composite compared with conventional composites: (**a**) optimal performance temperature; (**b**) outstanding mechanical properties. Reprinted with permission from Ref. [32]. Copyright 2016, Springer Nature.

Silicon carbide (SiC) has been widely used as a high-temperature ceramic below 1800 °C in recent decades, since it possesses the merits of structural stability, oxidation resistance, excellent mechanical properties, etc. [20,21,22,23]. In order to expand its application field at higher temperatures, ultrahigh-temperature ceramics (UHTCs) of transition metals (Zr, Hf, Ta, Hf, etc.) [24] with melting points over 3000 °C have been used in combination with SiC ceramics [25,26,27,28,29], which are referred to as Si-based ceramics. Recently, the ablation and oxidation behaviors of fiber-reinforced Si-based ceramic composites with different fiber structures as reinforcement, including whiskers, particles, and preforms, have been investigated [30,31]. Figure 1a,b detail the optimal performance temperature and outstanding mechanical properties of carbon fiber-reinforced Si-based ceramic composites compared with conventional composites [32]. However, the ablative mechanism of C/Si-based ceramic composites has not been comprehensively reviewed, constituting Si-based ceramics as the matrix and different fiber structures for reinforcement.

In this paper, the ablation and oxidation mechanisms, as well as the behaviors, of Si-based-ceramics coated or modified carbon fiber-reinforced composites, with different structures, are thoroughly reviewed. In Section 2, the preparation of C/Si-based composites is described. In Section 3, the ablation and oxidation behaviors, as well as the mechanisms, of carbon fiber-reinforced pure Si-based ceramic matrix composites (C/SiM composites, where C is carbon fiber, and M refers to B, C, N, etc.) are introduced. Section 4 provides a comprehensive review of the ablation and oxidation behaviors and mechanisms of transition metal Zr-supplemented C/SiM composites (C/SiZrM composites, where M refers to B, C, N, etc., typically ZrB_2_, ZrC, ZrB_2_, and ZrC). In Section 5, the ablation and oxidation behaviors, as well as the mechanisms, of C/SiM-Z composites are reviewed (Z refers to other transitional metals, i.e., Ta, Hf, Y, Ti, Mo, Cr, La, etc.). In these sections, the structures of the reinforced materials such as dispersive fibers, needle preforms, and 3D-braided performs are presented, and the methods of ablation or oxidation are also described. Lastly, Section 6 focuses on the challenges and future prospects in the development of carbon fiber-reinforced Si-based ceramic composites in order to promote their application fields. This paper can provide a reference for the preparation of anti-ablative composites, with explanations of their ablative mechanisms.

## 2. Preparation of C/Si-Based Composites

Ablation is an erosive phenomenon characterized by the removal of raw or oxidized materials through a combination of thermo-mechanical and thermo-physical as well as thermo-chemical factors resulting from high temperature, high pressure, and velocity of combustion flame. The primary methods to test the ablative or oxidation properties of the composites include plasma arc ablation, oxyacetylene flame ablation, etc. During ablation, the high heat flux of the combustion gas with high pressure and speed leads to chemical erosion and mechanical scouring, resulting ultimately in the damage and failure of composites. These factors are extrinsic elements impacting ablation mechanisms. Numerical simulation and evaluation system can also be established for diagnosing the flame during ablation [33].

The preparation process for C/Si-based composites is complex and expensive, mainly due to selection of reinforcement and the recombination of reinforcement and matrix. Carbon fiber has been widely utilized as a reinforcement material for composite structures in the aerospace field due to its high strength and high modulus as well as high melting point, etc. [34,35]. The development of textile technology has resulted in the existence of reinforced fiber in three primary forms. They are dispersive fibers, needled structure, as well as 2.5D or 3D structure with outstanding structural integrity. The third structure is commonly employed as high-reliability aircraft components and nose cones of missile warheads, as it can be woven into an integrated structure, and the preform can subsequently serve as reinforcements directly [36]. This structure has a more prominent ablative resistance than its 2D prefabricated counterpart. 

Moreover, Si-based ceramics have traditionally been used as the matrix of fiber-reinforced composites or as a protective layer for these composites to improve the ablative or oxidation resistance of the fibers [37]. Additionally, there are a variety of densification methods for preparing fiber-reinforced Si-based ceramics [38]. These mainly include hot pressing (HP), polycarbosilane infiltration pyrolysis (PIP), pressureless infiltration (PI), thermal gradient chemical vapor infiltration technique (TCVI), chemical vapor infiltration (CVI), chemical vapor deposition (CVD), pack cementation (PC), isothermal chemical vapor infiltration (ICVI), reactive melt infiltration (RMI), slurry infiltration (SI), etc. Typically, different methods are used together to improve the densification of composites. Preliminary investigations show that fiber-reinforced SiC-based composites, especially those with carbon fiber as reinforcement, are prone to forming cracks. This leads to carbon fibers becoming susceptible to oxidation through ingress of air when exposed to high temperatures. Therefore, a graphitic carbon interphase or BN interphase is applied to the surface of carbon fibers to create a weak bond between the fiber and the matrix, promoting the toughening behavior of the composite.

## 3. Ablation Behaviors and Mechanisms of Pure C/SiM Composites

Table 1 provides the ablation and oxidation properties of pure C/SiM composites in recent years, including preparing and ablation methods. Meanwhile, based on the different matrix, the corresponding ablative-resistance composites are classified into two types. These are, respectively, C/SiC with the same matrix of SiC or SiC coating, and C/Si_3_N_4_ with the same Si_3_N_4_ matrix. The ablation and oxidation mechanisms of C/SiC have been more extensively investigated because SiC represents a promising ablation inhibitor, owing to its effective specific weight and low cost. In subsequent sub-sections, the ablation and oxidation mechanisms of these composites are separately discussed in detail.

### 3.1. Ablation Behaviors and Mechanisms of C/SiC Composites

The ablation and oxidation mechanisms of C/SiC composites are similar. The carbon fiber can be protected by the outer interphase and matrix. These mechanisms are mainly centered on the outer materials. During the ablation process, the composites primarily undergo the following chemical reactions.

Reactions of carbon fiber or PyC:

(1) $$2C(s)+O_{2}(g)\to 2CO(g)$$

(2) $$C(s)+O_{2}(g)\to {CO}_{2}(g)$$

(3) $$C(s)+{CO}_{2}(g)\to 2CO(g)$$

Reactions of silicon carbide:

(4) $$SiC(s)+O_{2}(g)\to SiO(g)+CO(g)$$

(5) $$2SiC(s)+3O_{2}(g)\to 2SiO_{2}(l)+2CO(g)$$

(6) $$SiC(s)+3{CO}_{2}(g)\to SiO_{2}(l)+4CO(g)$$

(7) $$2SiC(s)+3O_{2}(g)\to 2SiO(g)+2{CO}_{2}(g)$$

(8) $$SiC(s)+2O_{2}(g)\to SiO(l)+{CO}_{2}(g)$$

Reactions of silicon dioxide and silicon monoxide:

(9) $$SiO_{2}(l)\to SiO_{2}(g)$$

(10) $$2SiO(g)\to SiO_{2}(s)+Si(s)$$

(11) $$SiO_{2}(s)+C(s)\to SiO(g)+CO(g)$$

**Figure 2:**
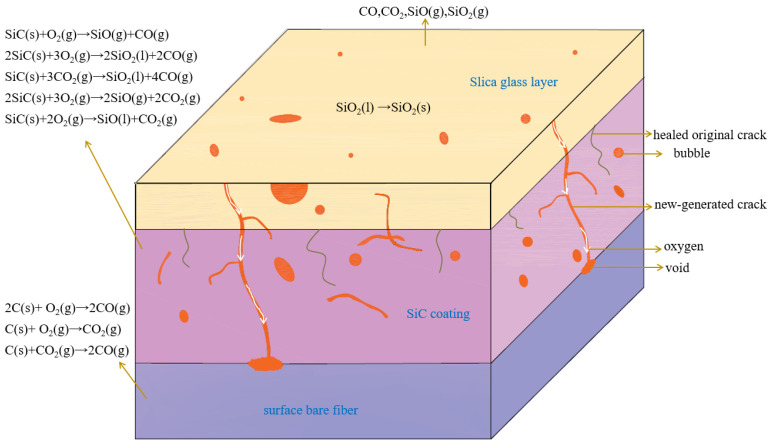
The graphical representation of the ablation progression in C/SiC composites and their protective mechanisms.

The ablation and oxidation behaviors of C/SiC composites are predominately controlled by oxidation, thermal decomposition, and sublimation, which are chiefly affected by the external ambient temperature [68,79]. Figure 2 illustrates the ablative progress and protective mechanism of C/SiC. Under a temperature of 1500 °C, oxygen reacts with SiC to yield SiO_2_. Below this temperature, SiO_2_ possessing a high fluidity, quickly seals any existing cracks of a certain width. The healed original cracks can be observed in the figure. In conjunction, the composites are shielded by solid or liquid SiO_2_, demonstrating effective ablative resistance performances without catastrophic repercussions. However, when the temperature surpasses 1500 °C, SiC oxidizes to generate gaseous SiO and CO. Concurrently, liquid SiO_2_ is easy to gasify and vaporize in abundance, leading to the creation of surface cracks. This allows oxygen to react with SiC, further permeating the coating thickness to form a penetrating crack. Consequently, the matrix loses its protective capacities over the fibers, with oxygen traveling to the fiber surface through these defects and inducing a reaction. As a result, gas holes, surface cracks, and skeleton structures are formed, initiating erosion of the composites [54].

**Figure 3:**
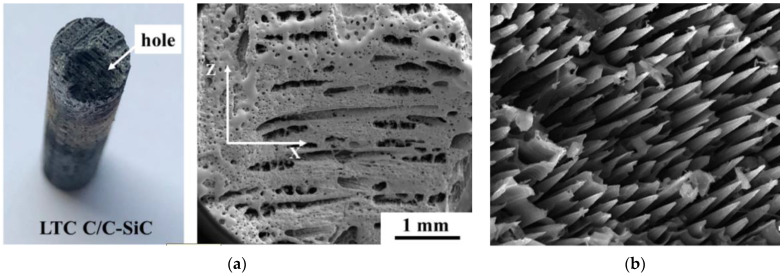
Ablation morphologies of C/C-SiC composite: (**a**) surface structures, reprinted with permission from [65], Copyright 2021, Elsevier; and (**b**) ablation center region, reprinted with permission from Ref. [67], Copyright 2021, Elsevier.

Moreover, ablation behaviors of C/SiC composites are also affected by the ablated method. When the composites undergo ablation in a plasma wind tunnel, both heat flux and stagnation pressure collectively control the ablation behaviors [52]. With low heat flux and stagnation pressure, SiO_2_ can deposit itself on the surface of the composites, causing minimal erosion and effectively protecting the fibers. In contrast, with high heat flux and stagnation pressure, SiC coatings are rapidly consumed due to sublimation and decomposition, resulting in quick exposure of fibers to plasma flow after the consumption of SiC coatings. When the composites are subjected to ablation under an oxyacetylene torch, the heat flux also affects the ablative behaviors [49]. The higher the heat flux, the faster the erosion rate of SiC, ensuing consequential defects in the matrix. As a result, the residual matrix shows reduced ablation resistance under mechanical denudation. In addition, these defects also loosen the surface, thereby enlarging the interface area between the oxidizing components and composites. Correspondingly, the oxidation rate would accelerate. The final ablative morphology of the composite can be observed in Figure 3, demonstrating obvious ablative characteristics and appearance of large surface ablation holes (Figure 3a). Simultaneously, the fibers in the central zone endure severe ablation and noticeable erosion at the fiber tip due to an extremely high temperature and heat flux.

Therefore, the ablation mechanisms of C/SiC composites are correlated not only with external ablative temperature, but also with the ablative method.

### 3.2. Ablation Behaviors and Mechanisms of C/SiBCN Composites

Compared with a traditional ceramic composite, during the ablation process, the BNC phase in a fiber-reinforced SiBCN composite can react with oxygen, thus generating B_2_O_3_ gas [80]. The evaporation of B_2_O_3_ gas can lower the surface temperature. Consequently, the B/Si ration in the glass decreases, with B being preferentially volatilized compared to Si, leading to an increase in the viscosity of the glass. Meanwhile, the low-viscosity B_2_O_3_ liquid can seal cracks and enhance the ablation resistance of composites, owing to its liquidity feature. Furthermore, in order to further study the effects of ultra-high temperature ceramics (UHTCs) as the secondary phase and SiBCN as the first phase in composites, it is imperative to discuss the basic ablation properties and mechanisms of fiber-reinforced SiBCN composites. It is found that, in addition to the reactions in Equations (1)–(11), the following chemical reactions of Equations (12)–(14) also occur during the ablation of the composites:

(12) $${SiO_{2}(l)+B}_{2}O_{3}(g)\to Borosilicateglass(l)$$

(13) $$4BN(s)+3O_{2}(g)\to {2B}_{2}O_{3}+2N_{2}(g)$$

(14) $$B_{2}O_{3}(l)\to B_{2}O_{3}(g)$$

**Figure 4:**
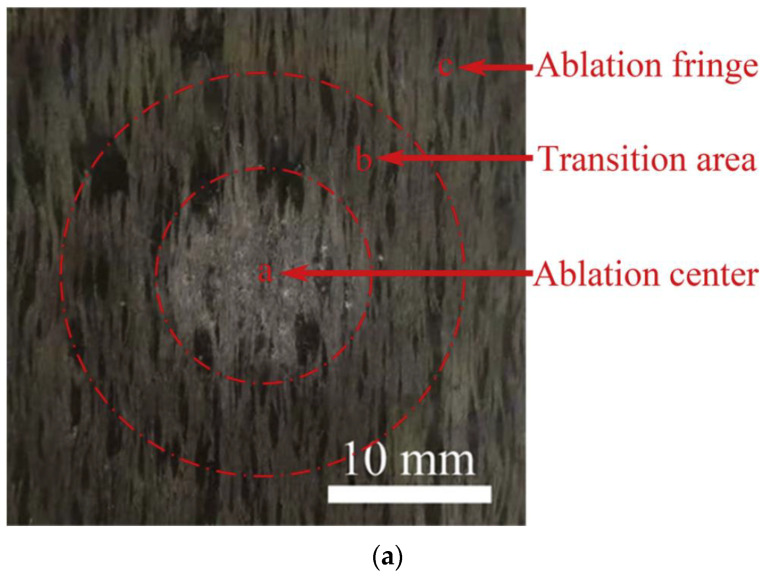

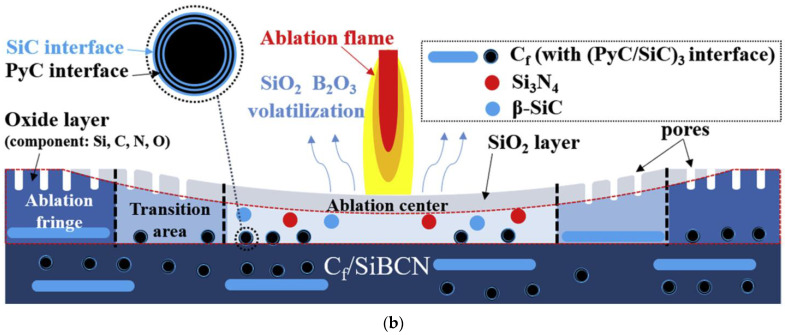
Schematic of (**a**) ablation surface and diagram of (**b**) mechanisms of C/(Pyc/sic)3SiBCN composites. Reprinted with permission from Ref. [78]. Copyright 2021, Elsevier.

Figure 4 provides the ablative surface and mechanisms of C/(Pyc/sic)_3_SiBCN composites [78]. The morphology of the ablation surface can be classified into the ablation center, transition zone, and ablation edge (Figure 4a). The ablative mechanism of fiber-reinforced SiBCN is related to the additional reaction of BN, as shown in Equation (13). The reaction yield of B_2_O_3_ can react with SiO_2_ to further form borosilicate glass. As the low-viscosity borosilicate glass diffuses, microcracks may be healed, significantly reducing the volatilization of B_2_O_3_, and enhancing the ablation resistance of composites. The volatilization of CO and N_2_ results in the appearance of variously sized bubbles on the ablated surface of the composite.

In addition to fiber-reinforced SiC or SiBCN matrix, composites with another matrix, such as Si_3_N_4_ ceramics with high strength, high thermal shock resistance, and good wear resistance are also used. During the ablation of the C/Si_3_N_4_ composite, carbon fiber is ablated in the central region, producing a large number of SiO_2_ droplets. Within the ring region, spherical solid SiO_2_ particles are formed, protecting the carbon fiber from further ablation [77]. 

In summary, the ablative mechanism of C/SiM composites primarily relies on a SiO_2_-rich layer protecting the fiber from ablation. Gas evolution happens sooner due to the higher volatility of boron-containing species. Additionally, the evaporation of gases can carry away energy and reduce the surface temperature―both processes inhibit oxidation and ablation within the composites.

## 4. Ablation of C/SiZrM Composites

Pure C/SiM composites can form a protective silica effectively at low temperature. It is difficult to meet the oxidizing and ablation atmosphere requirements above 2000 °C. SiC tends to oxidize and ablate at high temperatures (>1800 °C), coupled with chemical erosion. Hence, ultra-high temperature ceramics (UHTCs) have been introduced into C/SiM composites as the second phase of the matrix, encompassing many borides, carbides, and nitrides of early transition metals, particularly ZrB_2_ and ZrC. The addition remarkably improves the ablative resistance of composites at elevated temperature [81,82,83]. Table 2 gives the recent ablation and oxidation properties of C/SiZrM composites.

### 4.1. Ablation Behaviors and Mechanisms of C/ZrB_2_-SiC Composites

In order to improve the ablation-resistant and oxidation-resistant properties of C/SiC composites under complex circumstances and at elevated temperatures, ZrB_2_ is incorporated into the composite as the secondary phase of the matrix, which is called as C/ZrB_2_-SiC composites [160]. During the ablation process, the composites primarily undergo the following chemical reactions, aside from those outlined in Equations (1)–(11).

(15) $$2ZrB_{2}(s)+5O_{2}(g)\to 2ZrO_{2}(s)+2B_{2}O_{3}(l)$$

(16) $$2ZrB_{2}(s)+5O_{2}(g)\to 2ZrO_{2}(l)+2B_{2}O_{3}(g)$$

(17) $$ZrO_{2}(s)\to ZrO_{2}(l)$$

(18) $$ZrO_{2}(l)\to ZrO_{2}(g)$$

(19) $$ZrO_{2}(s)+{SiO}_{2}(l)\to ZrSiO_{4}(s)$$

**Figure 5:**
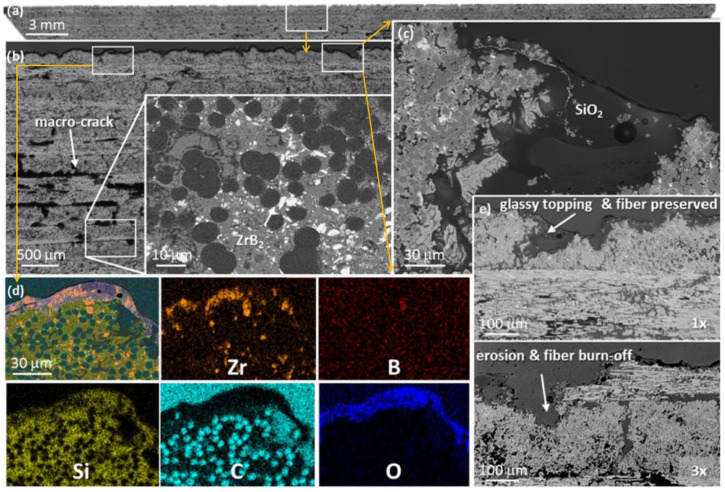
(**a**) Cross section of C/ZrB_2_-SiC composite after ablation; (**b**) magnified view of the boxed area in (**a**); (**c**) magnified view of the boxed area in (**b**); (**d**) EDS elemental map of the boxed area in (**b**); and (**e**) oxide evolution upon 1 (1×) or 3 (3×) sequential thermal attacks. Reprinted with permission from Ref. [35]. Copyright 2022, Elsevier.

**Figure 6:**
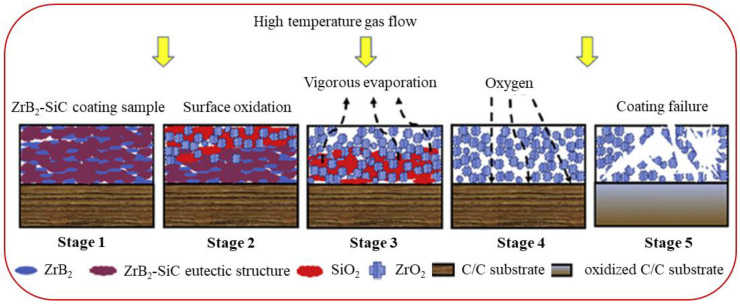
Schematic diagrams of C/ZrB_2_-SiC composite ablation process mechanism at 2000 °C. Reprinted with permission from Ref. [101]. Copyright 2020, Elsevier.

The ablation and oxidation behaviors of C/ZrB_2_-SiC composites are primarily affected by complex chemical erosion and mechanical denudation [86,161]. Figure 5 provides cross-section images of the C/ZrB_2_-SiC composite after ablation [35], illustrating the accumulation of a glassy layer on the outermost surface, which serves to protect the inner material. Figure 6 provides the detailed ablation mechanisms. In the ablative process, ZrB_2_ and SiC are oxidized to form SiO_2_, ZrO_2_, B_2_O_3,_ and borosilicate, the evaporation of which ultimately results in the porous surface layer. Between 450 °C and 1100 °C, these low viscosity and fluid B_2_O_3_ and borosilicate easily cover the external surface of the carbon fiber, forming a regular and dense oxidation scale. This is premised on the fact that the melting point of B_2_O_3_ is 450 °C and its boiling point 1850 °C. However, at higher temperatures, owing to the vapor pressure of the B_2_O_3_, a significant amount of B_2_O_3_ preferentially evaporates from the surface, forming an enriched SiO_2_ scale, where the melting point of SiO_2_ is 1670–1710 °C. Due to the lower oxidation temperature of ZrB_2_ and PyC, oxygen tends to diffuse inward through the oxide scale and reacts with these elements. In addition, the gradient of chemical potential and temperature within the composite encourages the oxidation of internal ZrB_2_. The formed ZrO_2_ provides a pinning effect, preventing the cracking and spalling of silica-scale glass. The formation of the SiO_2_-ZrO_2_ structure and ZrSiO_4_ glass can further obstruct oxygen diffusion and also have good adhesion to the fiber. Meanwhile, the formed gaseous B_2_O_3_ will migrate through the outer SiO_2_-rich scale layer. Therefore, the SiO_2_-rich oxide scale layer and a porous ZrB_2_-SiC-C_f_ layer are formed. As B_2_O_3_ evaporates, heat is dissipated and surface temperature of the composite decreases. With the diffusion of oxygen, the final products of SiO_2_, ZrO_2_, borosilicate glass, ZrSiO_4_ and continuous integrated SiO_2_-ZrO_2_-SiC ceramic layer prevent fiber structure from further ablation. Additionally, the escape of gaseous by-products, such as CO, CO_2_, SiO, and B_2_O_3_, produce a more pronounced thermal barrier effect.

### 4.2. Ablation Behaviors and Mechanisms of C/ZrC-SiC Composites

Refractory carbide ZrC, with its high melting point of 3540 °C, relatively low density (6.7 g/cm^3^), thermal shock resistance, and chemical inertness, etc., is regarded as an outstanding advanced ceramic, thus making it one of the most promising UHTCs. For this reason, it has been integrated into C/SiC composites and designed as C/ZrC-SiC composites suitable for application in extreme environments [162]. During the ablation process, the ablative performance of the C/ZrC-SiC composite is determined by a combination of chemical erosion, thermo-physical conditions, and mechanical denudation. Along with the reactions noted in Equations (1)–(11), there are other reactions that also occur in response to external environmental temperature when they are subjected to ablation.

(20) $$2ZrC(s)+3O_{2}(g)\to 2ZrO_{2}(s)+2CO(g)$$

(21) $$ZrC(s)+3CO_{2}(g)\to ZrO_{2}(l)+4CO(g)$$

(22) $$ZrC(s)+2O_{2}(g)\to ZrO_{2}(s)+CO_{2}(g)$$

(23) $$ZrO_{2}(s)\to ZrO_{2}(l)\to ZrO_{2}(g)$$

(24) $$SiO_{2}(s)\to SiO_{2}(l)\to SiO_{2}(g)$$

**Figure 7:**
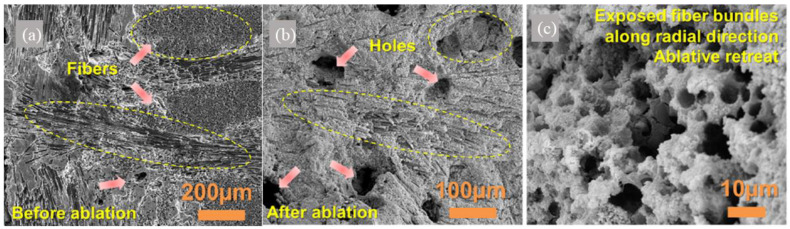
The ablation of the C/C-ZrC-SiC composite. (**a**) Before ablation; (**b**,**c**) after ablation. Reprinted with permission from Ref. [13]. Copyright 2022, Elsevier.

**Figure 8:**
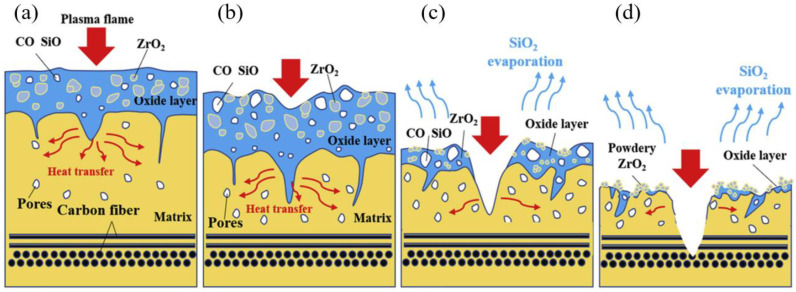
Ablation mechanisms of the C/ZrC-SiC composite. (**a**) Beginning; (**b**) dynamic equilibrium; (**c**) over evaporation; and (**d**) inward damage. Reprinted with permission from Ref. [145]. Copyright 2019, Elsevier.

Figure 7 depicts the ablation behaviors of the C/ZrC-SiC composite [13]. It is evident that the ZrC and SiC ceramics are evenly dispersed and sintered within closely braided carbon fibers. During the ablation of the composite, as shown in Figure 7b,c, intense oxidizing airflow persistently infiltrates through holes, exacerbating the oxidation reaction of ZrC and SiC, resulting in the gradual erosion and enlargement of the pores. Figure 8 presents the ablation mechanism of the C/ZrC-SiC composite. The combined ablative and oxidative behaviors of ZrC and SiC contribute to the self-healing feature of the composite. ZrC is the source of the refractory ZrO_2_ phase. The formed continuous liquid SiO_2_, SiO_2_-ZrO_2_ glassy layer, as well as ZrSiO_4_ act as effective barriers that obstruct the inward oxygen diffusion. The stable molten liquid ZrO_2_ scale can prevent the fiber from ablation when the temperature is above 2700 °C. It can cover and seal cracks as well as pores, hindering further in-depth oxygen diffusion into the oxidation-prone fiber. The singular ZrO_2_ layer features a weak interfacial bond and can easily fall off. However, the glassy silica phases can permeate the gaps in the ZrO_2_ skeleton, stick to the central ZrO_2_ layer, and facilitate the sintering of porous ZrO_2_, consequently strengthening its intact surface. Simultaneously, the formed glassy ZrO_2_-SiO_2_ layer is generated on the surface, and a porous interlayer is formed by the ZrO_2_ skeleton and a few silica glasses, which is due to the evaporation of CO, CO_2_, SiO, and SiO_2_. The ZrO_2_-melting layer, the porous layer, and SiO_2_-rich layer together constitute the comprehensive glassy ZrO_2_-SiO_2_, which inhibits the erosion of oxidative gas. Moreover, the formation of continuous integrated SiO_2_-ZrO_2_-ZrC-SiC layer safeguards the C/C preform from further ablation by acting as a thermal and oxygen diffusion barrier [114].

### 4.3. Ablation Behaviors and Mechanisms of C/ZrB_2_-ZrC-SiC Composite

To further improve the ablation resistance of the C/SiC composite at elevated temperatures, UHTCs of ZrB_2_ and ZrC can be collectively incorporated into the composite due to their high melting points of 3250 °C and 3540 °C, along with their low densities of 6.1 g/cm^3^ and 6.7 g/cm^3^, respectively, which will create a C/ZrB_2_-ZrC-SiC composite [163]. Compared to C/ZrC-SiC and C/ZrB_2_-SiC composites, this incorporation can yield better hardness, fracture toughness, and flexural strength. Moreover, Equations (1)–(24) will occur during ablation.

**Figure 9:**
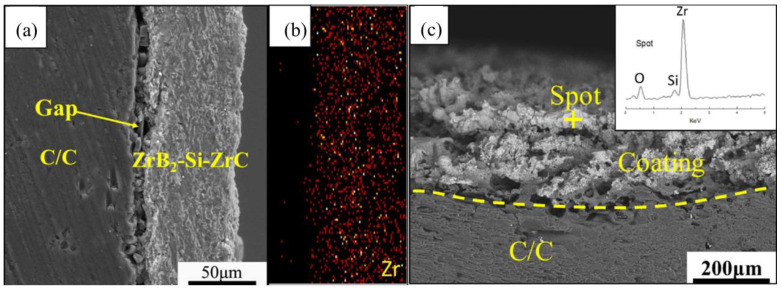
The ablation of the C/ZrB_2_-ZrC-SiC composite. (**a**) Before ablation; (**b**) the distribution of Zr element before ablation; and (**c**) after ablation. Reprinted with permission from Ref. [155]. Copyright 2018, Elsevier.

**Figure 10:**
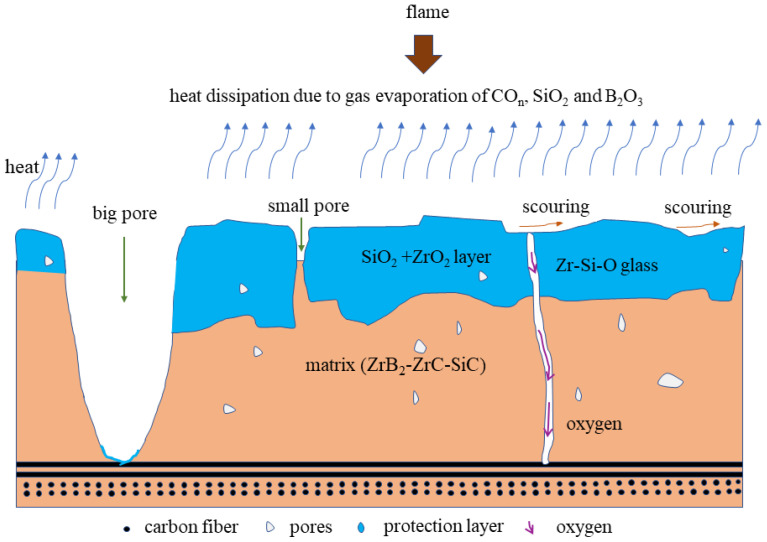
Ablation mechanism of the C/ZrB_2_-ZrC-SiC composite.

Figure 9 details the morphology of the C/ZrB_2_-ZrC-SiC composite both before and after ablation, with a relatively uniform Zr element (Figure 9b). The vaporization of SiO escape promotes the development of poriferous and lax structure (Figure 9c). The ablation mechanisms of the C/ZrB_2_-ZrC-SiC composite are displayed in Figure 10. The ZrB_2_-ZrC-SiC matrix undergoes oxidation to form molten oxide scales of ZrO_2_-SiO_2_, thus developing a Zr-Si-O glass phase, which possesses high viscosity. This can flow and seal the pores on the ablated surface, meanwhile most of the oxidation product B_2_O_3_ evaporates above 1650 °C. Concurrently, the evaporation and fusion of gases (CO_n_, SiO_2_ and B_2_O_3_) can dissipate the surface heat of the substrate. Resultantly, many small pores are formed in the glass layer owing to the gas diffusion and evaporation, while large pores are formed as a result of matrix ablation and possibly pre-existing pores before ablation. Therefore, oxygen diffuses into the interior via these channel pores. In addition, both the matrix and the molten oxidation product can be stripped away by a high-velocity and high-pressure flame [148]. The ablation of the C/ZrB_2_-ZrC-SiC composite predominantly rests on the oxidation process and the mechanical ablation triggered by the flame.

## 5. Ablation of the C/SiZM Composites

To further improve the ablative resistance of C/SiM composites in complex and extreme circumstances, other transition metals except from Zr, such as Ta, Hf, Y, Ti, Mo, Cr, La, etc., are also incorporated into the C/SiM composite [164,165,166,167,168], which is called a C/SiZM composite (Z=Ta, Hf, Y, Ti, Mo, Cr, La, etc.). Table 3 showcases recent ablation and oxidation properties of C/SiZM composites and provides a summary of both historical and recent ablation research results of C/C-SiC-Z composites.

Composites of C/SiM modified with Hf demonstrate remarkable thermal stability, where the melting point of Hf approaches 2227 °C. During the ablation process, the following chemical reactions take place:

(25) $$2HfC(s)+3O_{2}(g)\to 2HfO_{2}(s)+2CO(g)$$

(26) $$HfC(s)+O_{2}(g)\to HfO(g)+CO(g)$$

(27) $$HfC(s)+2O_{2}(g)\to HfO_{2}(s)+CO_{2}(g)$$

(28) $$2HfB_{2}(s)+5O_{2}(g)\to 2HfO_{2}(s)+2B_{2}O_{3}(g)$$

**Figure 11:**
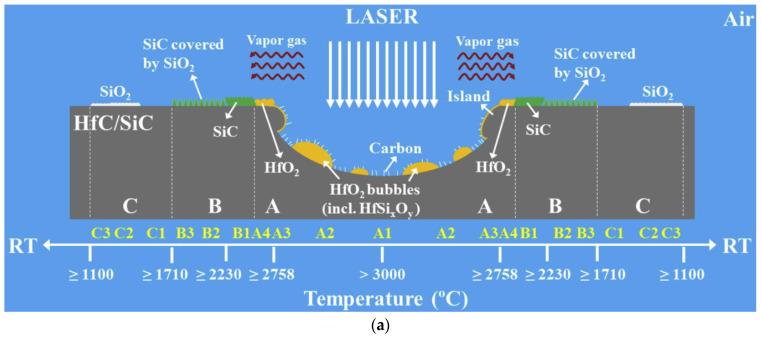

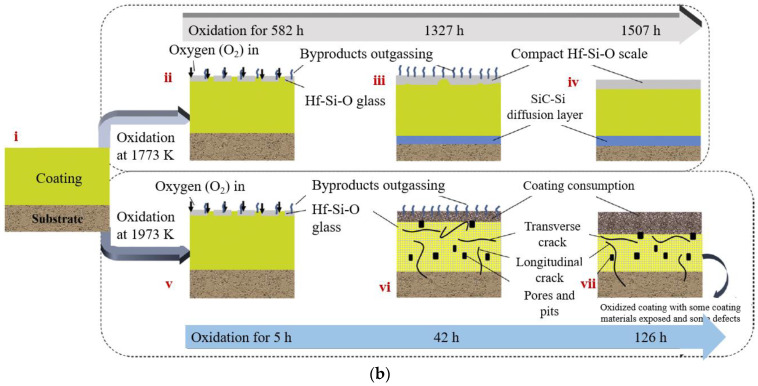
Ablation mechanisms of the (**a**) C/SiC-HfC, A,B,C refer to the center region, transitional region and fringe region, respectively, reprinted/adapted with permission from Ref. [171], Copyright 2019, Elsevier; and (**b**) C/SiC-HfB_2_ composite, ⅰ,ⅱ,ⅲ,ⅳ denote the oxidation process occurring at 1773 K over time, while ⅰ,ⅴ,ⅵ,ⅶ signify the same process at 1973 K as time progresses, reprinted with permission from Ref. [177], Copyright 2021, Elsevier.

The ablation mechanisms of the C/SiC-HfC and C/SiC-HfB_2_ are provided in Figure 11. The formation of SiO_2_-HfO_2_ protects the fiber from ablation during the initial ablation. However, these ablative products lose their protective function over time owing to mechanical denudation and thermal chemical ablation damage. In fact, HfO_2_, SiHf_x_O_y_-based layers (SiHf-O glass) and liquid SiO_2_ can protect the fiber from ablation.

In terms of the ablative mechanisms of the Ta-added C/SiM composite, the formation of a mosaic-structured Ta-Si-O glassy layer, alongside the SiO_2_ layer and Ta_2_O_5_ on the surface of the C/C composite, inhibits oxides from damaging the fibers. Ta_2_O_5_, acting as “pinning phases”, is beneficial to maintain the stability of TaB_2_-SiC coating and augmenting its ablative resistance. In the case of the Zr-La added C/SiM composite, the oxide of La promotes the liquid phase sintering of ZrO_2_, and generates a molten phase of La_2_Zr_2_O_7_. Additionally, evolution of La_2_O_3_, La_2_Si_2_O_7_, La_0.71_Zr_0.29_O_1.65_, and micron-sized ZrO_2_-La_2_O_3_-SiO_2_ liquid phase layers provide superb oxygen barrier protection for the composites. When Zr-Hf is incorporated into C/SiM composites, the consequent dense, compact, and continuously oxidized HfO_2_-ZrO_2_-SiO_2_ mixture layer is helpful for ablation protection. Moreover, the addition of Zr-Mo to the C/SiM composite leads to the formation of SiO_2_-ZrO_2_-Mo_4.8_SiC_0.6_ oxide protective barrier that impedes oxygen diffusion into the substrate interior. For the Ta-Hf modified C/SiM composite, an integral scale constituted by Hf-Ta-Si-O (HfO_2_-Ta_2_O_5_-SiO_2_ ceramic sheet) oxides act as oxygen insulator, and the formation of micro-cracks mitigates thermal stress. For the Zr-Ti enhanced C/SiM composite, the multiphase oxidation scale of Zr-Ti-Si-O glass provides exceptional resistance against the ablation of the substrate.

As for other Ti, Y, Cr, Al, V, Mo, Sm, Cu, Nd, Nb, et al., added C/SiM composites, the primary protective oxide layers TiO_2_, Y_2_O_3_, Cr_2_O_3_, Al_2_O_3_, V_2_O_3_ (V_2_O_5_), MoSi_3_ (Mo_5_Si_3_), Sm_2_O_3_, CuxO, Nd_2_O_3_, NbO (NbO_2_ and Nb_2_O_5_) contribute to the anti-ablation resistance of the substrate.

## 6. Conclusions and Future Perspectives

In this paper, ablation characteristics of carbon fiber-reinforced Si-based composites has been exhaustively reviewed. The ablation mechanisms were comprehensively provided. For the ablation of carbon fiber-reinforced Si-based materials, oxides of Si and other UHTCs (Zr, Ta, La, Hf, Mo, Ti, Y, Cr, Al, V, Mo, Sm, Cu, Nd, Nb, et al.) with high melting points can collaboratively protect the carbon fiber substrate from ablation, particularly at elevated temperatures. The synergistic effect of SiO_2_ combined with the corresponding oxides of UHTCs can potentially extend their usage to environments with higher temperatures. In addition, over time, the gas oxidations gradually evaporate and a large number of pores and cracks are formed on the surface. Consequently, the oxygen diffuses into the carbon fiber substrate and causes oxidation. Ultimately, this results in the damage of the composites. Meanwhile, thermo-mechanically, this can also result in the depletion of surficial coating.

However, following issues need further attention in the study of carbon fiber-reinforced high-temperature ceramic composites to enhance their practical applications:

(1) The mechanical properties

When high-temperature materials are utilized in actual environments, consideration must be given not only to their anti-ablation properties, but also to their mechanical properties. Therefore, properties such as tension, compression and bending, etc. should all be studied concurrently to provide an accurate assessment of their comprehensive performance in the future.

(2) Selection of reinforcement

In order to further optimize performance under high-temperature conditions for certain materials, an appropriate reinforcement structure can be reasonably selected. When they are used as the primary structural component, 2D or 3D preforms with an integral structure can be employed directly.

(3) Matrix modification

The use of a Si-based matrix is selected primarily due to the formation of glassy and molten SiO_2_ at temperatures approaching to 1800 °C. Coincidentally, when the temperatures exceed this figure, refractory metals can also be incorporated. This would result in the production of a Si-relevant oxide together with other refractory metals. As a result, the more stable and continuous protective dense oxide scale can be created, limiting the diffusion pathways, and ensuring the structural stability of the composites. Simultaneously, the metal size and ratios used should also be considered, as different ratios can produce different reaction products, and hence further affect the overall protective capacities of the composite. 

Finally, further research is required to understand which type of refractory materials can offer the best oxidation resistance for the composite, and what kind of test methods are best to evaluate the anticipated resistance abilities for their intended applications.

## Figures and Tables

**Table 1:** Materials, methods, and ablation and oxidation properties of pure C/SiM composites.

Composites	Main Structure	Interphase	Matrix (Coating)	Preparing Method	Ablation Type	Mass Ablation Rate (mg∙s^−1^)	Liner Ablation Rate (mm∙s^−1^)	Ref.
C/SiC	4D axes carbon fiber preform		SiC coating		arcplasma torch	3	0.1311	[39]
	3D braid carbon		SiC coating		isothermal oxidation			[40]
	2D C/C needle	PyC	SiC	ICVI; PI;	engine torch			[41]
	carbon fiber (M30)	PyC	SiC	CVI	oxyacetylene torch			[42]
	3D C orthogonal structure		SiC	PIP, HP	oxyacetylene torch			[43]
	3D braid C/C	PyC	SiC	CVI, CVD	gas mixture (O_2_/H_2_O/Ar)			[44]
	3D needled (30 vol%)	PyC	SiC	CVI; LSI	oxyacetylene torch	1.6	0.0039	[45]
	3D braided	PyC	SiC	PIP	oxyacetylene torch			[46]
	2D plain woven carbon- fabric	PyC	Ph/SiC	LSI	oxyacetylene torch	1837		[47]
	needle punched disk felts	PyC	SiC	PIP; TCVI	oxyacetylene torch	1.53		[48]
	2.5D carbon fiber felts	PyC	SiC	PIP; TCVI	oxyacetylene torch			[49]
	2.5D needle puncher felts	PyC	SiC coating	TCVI; PC	oxyacetylene torch			[50]
	needle-carbon fiber felts	PyC	SiC	CVI; molten infiltration	oxyacetylene torch			[51]
	3D needled felt (T300)	PyC	SiC coating	CVI	plasma wind tunnel			[52]
	4D woven carbon preforms		SiC	impregnation	UH25 was used as fuel; N_2_O_4_ as oxidizer		0.005	[53]
	carbon fiber	PyC	SiC coating	CVD; ICVI	oxyacetylene torch			[54]
	2D C/C	PyC	SiC coating	slurry and sintering	isothermal			[55]
	2D C/C needle	PyC	SiC	CVI	hypersonic flowing propane flame			[56]
	2.5D needle punched carbon fiber felt	PyC	SiC	PI; PIP; CVI	plasma generator equipment		0.017	[57]
	2D carbon fiber	BN	SiC	CVD	temperature programmed oxidation			[58]
	carbon fibers (T-300)		SiC/SiC coating	PIP; CVD	air			[59]
	carbon fiber plain fabrics		ph/silicon	LPI	thermal plasma torch			[60]
	3D needle preform	PyC	SiC and Si	CVI; CVD	oxyacetylene torch	6.2		[61]
	carbon fiber	PyC	SiC	CVD	wind-tunnel			[62]
	carbon fiber cloth	PyC	SiC nanowires	CVD	oxyacetylene torch	0.400		[63]
	carbon preform	graphitized	SiC		oxy-kerosene hypersonic torch	0.09		[64]
	3D preform	PyC	SiC	CVI; PIP	plasma arc ablation	0.56	1.1 × 10^−4^	[65]
	2.5D preform	PyC	SiC		millisecond laser			[66]
	2D carbon fiber felts		SiC	CVI;	plasma stream			[67]
	carbon fiber	PyC	SiC coating	PC	furnace			[68]
	carbon fiber		SiC	CVI	continuous wave lasers			[69]
	2.5D needle punched preform	PyC	SiC	CVI; PIP	plasma generator equipment	0.133	0.0141	[70]
	3D needle-punched preform	PyC	SiC	CVI; PIP	plasma wind tunnel			[71]
	needled preform of carbon felt	PyC	SiC	PIP	oxy-acetylene torch			[72]
	2.5D carbon fiber felt		PyCx-SiCy	CVI;	oxyacetylene torch		0.0016	[73]
	carbon structure		PyCx-SiCy	CVI	oxyacetylene torch		0.0013	[74]
	2.5D needle punched preform	PyC	SiC coating	CVI; PC	oxyacetylene torch	0.0001	0.0003	[75]
	carbon fibre needled felts	PyC	SiC	RMI; CVI	oxyacetylene torch	0.75		[76]
C_f_/Si_3_N_4_	needle preform		Si_3_N_4_	LPCVI; CVI	oxyacetylene torch			[77]
C_f_/SiBCN	3D needled carbon fiber preform	(PyC/SiC)_3_	SiBCN	CVI; PIP	plasma ablation flame	0.0427	0.0017	[78]

**Table 2:** Materials and methods, as well as ablation and oxidation properties of C/SiZrM composites.

Composites	Main Structure	Interphase	Matrix (Coating)	Preparing Method	Ablation Method	Mass Ablation Rate (mg∙s^−1^)	Liner Ablation Rate (mm∙s^−1^)	Ref.
C/ZrB_2_-SiC	2D plain woven carbon cloth	PyC	ZrB_2_-SiC	CVI; SP	oxyacetylene torch			[84]
	needle punched carbon fiber webs	PyC	ZrB_2_-SiC	CVI; HCVI	arc-heated wind tunnel			[85]
	2D needle punched carbon fiber preform	PyC	ZrB_2_-SiC	CVI	arc-heated wind tunnel			[86]
	2D C/C composites		ZrB_2_-SiC	SAPS	oxyacetylene torch		1.7 × 10^−4^	[87]
	2D C/C composites		ZrB_2_-SiC	pack-cementation	oxyacetylene torch	0.062	0.0044	[88]
	needle punched integrated felt	PyC	ZrB_2_-SiC	TCVI; PIP	oxyacetylene torch			[89]
	2D C/C composites		ZrB_2_-SiC	SAPS; PC; SI	oxyacetylene torch			[90]
	2D needled carbon fiber preform		ZrB_2_-SiC	slurry-sintering; CVR	plasma generator			[91]
	needle-punching carbon fiber preform		ZrB_2_-SiC	TCVI; PIP	oxyacetylene torch			[92]
	needled integrated preform		ZrB_2_-SiC	pressing, pyrolysis; RSI	oxyacetylene torch	1.3		[93]
	C/C composites	PyC	SiC-ZrB_2_	CVD; CVI	oxyacetylene torch			[94]
	3D braided C/SiC preform		ZrB_2_-SiC	painting slurry; CVD; PIP	oxyacetylene torch	22.9	0.0236	[95]
	2D SiC-coated C/C preform		ZrB_2_-SiC	TCVI; PC; SAPS	oxyacetylene torch			[96]
	2D SiC-coated C/C preform		ZrB_2_-SiC-Si	PC	oxyacetylene torch	1.5	0.00021	[97]
	3D braided SiC-coated C/C preform		ZrB_2_-SiC	CVD; slurry painting; PIP	oxidation in air			[20]
	carbon fiber		ZrB_2_-SiC	slurry infiltration; HP	homemade testing chamber			[16]
	short random/aligned continuous carbon fiber		ZrB_2_-SiC	HP; SPS	arc-jet plasma			[35]
	porous C/C preform		SiC-ZrB_2_	RMI; ICVI	oxyacetylene torch	0.61	0.00672	[98]
	short carbon fiber		phenolic-ZrB2-SiC		oxyacetylene torch	14	0.000168	[99]
	needle-punched carbon preform	PyC	ZrB_2_-SiC-Si	CVI	oxyacetylene torch			[100]
	C/C preform		ZrB_2_-SiC	HPPS	oxyacetylene torch	2.46		[101]
	2D C/SiC preform		ZrB_2_-SiC	CVI; CVD	oxyhydrogen torch			[102]
	PAN-based carbon fiber	PyC	ZrB_2_-SiC	PIP	arc-jet wind tunnel			[103]
	C/C carbon fabric		ZrB_2_-SiC	LSI;	oxyacetylene torch		217	[104]
C/ZrC-SiC	3D 4-directional carbon fiber preform		ZrC-SiC	CVD; PIP	oxyacetylene torch	0.69	0.026	[79]
	3D 4-directional carbon fiber preform		ZrC-SiC	PIP	plasma wind tunnel	0.7	0.0009	[105]
	3D needle-punched carbon fabrics	PyC	ZrC-SiC	CVI; SI; RMI; PIP	plasma wind tunnel			[106]
	2D C/C carbon felts		ZrC-SiC	ICVI; RMI	oxyacetylene torch	0.24	0.00133	[107]
	2D C/C carbon felts		ZrC-SiC	ICVI; RMI	oxyacetylene torch	0.21	0.00144	[108]
	2D needled C/C carbon fiber felts		ZrC-SiC	TCVI; PIP	oxyacetylene torch	0.40	0.00102	[109]
	porous C/C preform		ZrC-SiC	PIP	oxyacetylene torch	2.29	0.0003	[110]
	2.5D carbon fiber felts	PyC	ZrC-SiC	TCVI; PIP	oxyacetylene torch	1.9	0.012	[111]
	2.5D needled carbon felts	PyC	ZrC-SiC	TCVI; PIP	oxyacetylene torch	0.585	0.00133	[112]
	2.5D needled integral C/C preform		ZrC-SiC	CVD; RMI	oxyacetylene torch	0.02	3.3 × 10^−4^	[113]
	2.5D needled C/C felts		ZrC-SiC	CVI; PIP	plasma generator	1.57	3.7 × 10^−4^	[114]
	3D orthogonal braided carbon fiber preform	PyC	ZrC-SiC	CVI; RMI	oxyacetylene torch			[115]
	2D needled C/C perform		ZrC-SiC	CVI; PIP; RMI	plasma generator	2.6	3.7 × 10^−3^	[116]
	C/C preform		ZrC-SiC	RMI; PIP	plasma generator	0.0045	4.8 × 10^−3^	[117]
	3D braided carbon fibers		SiC/Zr-Si-C/SiC	PIP; CVD	oxyacetylene torch	27.4	0.0255	[118]
	3D carbon fiber preform		ZrC-SiC	CVD; PIP	oxyacetylene torch			[119]
	2D needled C/C felts		ZrC-SiC	PIP	oxyacetylene torch	37.5	2.48 × 10^−3^	[120]
	C/C felt preform		SiC-ZrC	CVI; PIP	Developed personally		3 × 10^−3^	[121]
	needled carbon fiber integer preform		ZrC-SiC	CVI; PIP	plasma flame	1.73	1.94 × 10^−4^	[122]
	porous needling C/C preform		SiC-ZrC	RMI; CVI	oxyacetylene flame	1.18	2.47 × 10^−3^	[123]
	3D needle-punched carbon fiber fabrics		SiC-ZrC	slurry impregnation; CVI	arc-heated air plasma		0.039	[124]
	2D needle-punched carbon felt	PyC	SiC-ZrC	CVI; PIP; ICVI; TCVI	oxyacetylene torch	2.95	0.015	[125]
	C/C preform		SiC-ZrC	RMI; ICVI	oxyacetylene torch	1.21	5.9 × 10^−3^	[126]
	C/C preform		SiC-ZrC	CVI; PIP	oxyacetylene torch			[127]
	needle-punched carbon felt	PyC	SiC-ZrC	ICVI; PIP; ECVI	oxyacetylene flame	0.04	3.7 × 10^−4^	[128]
	2.5D carbon fiber preforms	PyC	ZrC-SiC	CVI	oxyacetylene torch	0.147	9.8 × 10^−3^	[129]
	2D needled carbon fiber preform	PyC	ZrC-SiC	TCVI	oxyacetylene flame	0.298	8.2 × 10^−4^	[130]
	needled carbon felt		ZrC-SiC	CVI; PIP	plasma generator	0.558	0.01633	[131]
	2D needled carbon felts	PyC	ZrC-SiC	CVI; PIP	oxyacetylene torch	0.46	6.7 × 10^−4^	[132]
	needled felt-structured C/C preform		SiC-ZrC	RMI	oxyacetylene torch	0.29	2.48 × 10^−3^	[133]
	C/C preform		ZrC-SiC	liquid sintering; RIM	oxyacetylene torch	0.87	2.8 × 10^−4^	[134]
	C/C preform		ZrC-SiC	RIM	oxyacetylene torch	0.8	3.85 × 10^−3^	[135]
	T300 fiber cloth		ZrC-SiC	PIP	laser ablation		0.0748	[136]
	2D C/C preform		SiC/ZrC-SiC		oxyacetylene flame	1.2		[137]
	carbon felts		SiCnw/PyC/ZrC-SiC	CLVD	oxyacetylene torch	0.47	7.3 × 10^−4^	[138]
	2.5D needling carbon felt		ZrC-SiC	CLVD; PIP	oxyacetylene flame	1.22	1.07 × 10^−3^	[139]
	2.5D needled carbon fiber felts		ZrC-SiC	CLVD	oxyacetylene torch	0.39	5.2 × 10^−4^	[140]
	2D carbon fiber cloths	PyC	SiC-ZrC	CVI	oxyacetylene ablator	1.17	7.5 × 10^−3^	[141]
	2D needled C/C preform		SiC-ZrC	CVI	oxyacetylene torch	0.29	4.2 × 10^−4^	[142]
	2D needle-punched C/C preform		SiC-ZrC	PC	oxyacetylene flame	1.378	1.928 × 10^−3^	[143]
	3D carbon fiber	PyC	SiC-ZrC	CVD	oxyacetylene flame			[13]
	3D needle-woven carbon fiber felt		SiC-ZrC	CVI	oxyacetylene torch	7.1	4.7 × 10^−3^	[144]
	3D needle- carbon fiber felt	PyC-SiC	SiC-ZrC	CVI; RMI	plasma torch			[145]
	2.5D needled C/C preform		ZrC/SiC	CVD	oxyacetylene torch	0.84		[146]
	2D needle-punched carbon felts	PyC	ZrC-SiC	CVI	oxyacetylene torch	0.343	4.67 × 10^−4^	[147]
C/ZrB_2_-ZrC-SiC	3D carbon fiber preform	PyC	ZrB_2_-ZrC-SiC	CVI; PIP	oxyacetylene; plasma torch	0.5; 0.13	1 × 10^−3^; 4 × 10^−5^	[81]
	needled C/ZrB_2_ preform		ZrC-SiC	vacuum impregnation; PIP	plasma generator	5.09	2.61 × 10^−3^	[148]
	2D C/C preform		ZrB_2_-ZrC-SiC	CVD; PC; SAPS	oxyacetylene torch	0.23	6.5 × 10^−5^	[149]
	2D needle punched carbon fiber fabric	PyC	SiC-ZrB_2_-ZrC	TCVI; PIP	oxyacetylene torch			[150]
	needle punched carbon fiber felts	PyC	SiC-ZrB_2_-ZrC	PIP; TCVI	oxyacetylene torch			[151]
	2D carbon fiber reinforcement felts	PyC	SiC-ZrB_2_-ZrC	CVI; PIP	oxyacetylene torch	0.0252	4.15 × 10^−4^	[152]
	carbon felts	PyC	ZrB_2_-ZrC-SiC	TCVI; PIP	oxyacetylene torch			[153]
	pitch-based carbon fibers		ZrB_2_-ZrC-SiC	HP	oxyhydrogen torch			[14]
	plain weave carbon fiber		ZrB_2_-SiC-ZrC	Silicon melt-infiltration	oxyhydrogen torch			[15]
	2.5D needle punched carbon fiber fabric		SiC-ZrB_2_-ZrC	TCVI; PIP	plasma and compressed air			[25]
	Carbon fiber cloth		ZrB_2_-SiC/ZrC	HP	oxyhydrogen torch	2.8		[154]
	2D C/C preform		ZrB_2_-SiC-ZrC	SAPS; RMI	oxyhydrogen torch	0.016	1.3 × 10^−3^	[155]
C/SiC-ZrSi_2_	3D needled carbon felts	PyC	SiC-ZrSi_2_	CVI; RMI	oxyacetylene torch			[156]
C/Zr_2_Si	3D needled carbon fiber felts	PyC	Zr_2_Si	RMI; CVI; arc melting	economical laser beam			[157]
C/SiC-Si-Zr	3D needled carbon fiber felts	PyC	SiC-Si-Zr	RMI; CVI	economical laser beam		0.0407	[158]
C/SiC/ZrO_2_	carbon fabric		Ph/SiC/ZrO_2_	ball milling	oxyacetylene flame	70.848	0.031	[159]

**Table 3:** Materials, methods, ablation, and oxidation properties of the C/SiZM composites.

Added UHTC	Composites	Main Structure	Interphase	Matrix (Coating)	Preparing Method	Ablation Method	MR * (mg∙s^−1^)	LR * (mm∙s^−1^)	Ref.
Si-Hf	C/SiC-HfC	2D needled C/C felts		SiC-HfC	PIP; TCVI	oxyacetylene torch	2.5	1.2 × 10^−4^	[169]
		3D needle-punched felt	PyC	SiC-HfC	CVI; RMI	plasma wind tunnel			[170]
		2D carbon fabrics		SiC-HfC	SPS; PIP	CO_2_ laser	12.6		[171]
		C/C-HfC-SiC		SiC and HfC coating	CVR; VPS	ICP plasma wind tunnel			[172]
	C/C-HfB_2_-SiC	SiC-coated C/C preform		HfB_2_-SiC	PC; in situ reaction	oxyacetylene	0.147	2.67 × 10^−4^	[173]
	C/C-SiC-HfC	2.5D C/C preform		SiC-HfC	in situ reaction; CVD	oxyacetylene	2.05	1.93 × 10^−3^	[174]
	C/SiC-HfC	3D needle-punched preforms	PyC	SiC-HfC	CVI; RMI; PIP	oxyacetylene torch	1.5	4 × 10^−3^	[175]
	C/C-HfB_2_-SiC	2.5D needled carbon fiber felts	PyC	HfB_2_-SiC	CVI; PIP; HSLSI	oxyacetylene flame	0.5	4.15 × 10^−4^	[176]
	C/C-SiC-HfB_2_-Si	2.5D C/C preform		SiC-HfB_2_-Si	SP; GSI	oxyacetylene flame	0.07	7.2 × 10^−4^	[177]
	C/C-SiC-HfC	C/C preform		(SiC-HfC)_4_/SiC	LPCVD;	oxyacetylene torch	0.64	5.3 × 10^−4^	[178]
	C/SiHfBCN	2D carbon fabric		SiHfBCN	PIP	CO_2_ laser beam			[179]
	SiC_f_/HfC-SiC	2.5D SiC preform	PyC	HfC-SiC	CVI; PIP	oxyacetylene torch	1.32	7.37 × 10^−3^	[180]
Si-Ta	C/TaB_2_-SiC	2D-C/C preform		TaB_2_-SiC	PC; TCVI	oxyacetylene torch		4.2 × 10^−3^	[181]
	C/TaSi_2_	3D carbon fiber preform		TaSi_2_	pressure filtration	plasmatron			[182]
	C/SiC_nw_-TaSi_2_	carbon fiber preform		SiC_nw_-TaSi_2_	rapid sintering	oxyacetylene torch			[12]
	C/C-SiC-TaSi_2_	2D SiC-coated C/C preform		SiC-TaSi_2_	SAPS; PC	oxyacetylene torch	0.4	9 × 10^−4^	[183]
	C/C-SiC-TaC	needle-integrated C/C felts	PyC	C-SiC-TaC	CVI	oxyacetylene flame			[184]
Si-Zr-La	C/C/-ZrC-SiC-LaB_6_	2D C/C preform		ZrC-SiC-LaB_6_	SPS; SAPS	oxyacetylene torch			[185]
	C/C-SiC-ZrC-La	2D C/C preform		SiC-ZrC-La	PC; SAPS	oxyacetylene torch			[186]
	C/C-SiC-ZrB_2_-LaB_6_	3D C/C preform	PyC	SiC-ZrB_2_-LaB_6_	PIP; CVI;	plasma generator	0.38	3.7 × 10^−4^	[187]
	C/C-SiC-ZrB_2_-La_2_O_3_	2D C/C preform		SiC-ZrB_2_-La_2_O_3_	PC; SAPS	oxyacetylene flame	0.558	1.67 × 10^−5^	[188]
	C/C-ZrB_2_-SiC-La_2_O_3_	2D carbon fiber plain cloth	PyC	ZrB_2_-SiC-La_2_O_3_	CVI; SI; PIP	air plasma flame			[28]
	C/C-SiC-ZrC-La_2_O_3_	2D C/C preform		SiC-ZrC-La_2_O_3_	SAPS	oxyacetylene torch			[189]
Si-Zr-Hf	C/C-SiC-HfC-ZrC	2D C/C preform		SiC-HfC-ZrC	TCVI; PIP	oxyacetylene torch			[190]
	C/HfC-ZrC-SiC	2.5D needled C/C preform		HfC-ZrC-SiC	RMI	oxyacetylene torch	1.5	1.1 × 10^−3^	[191]
	C/C-HfC-ZrC-SiC	3D C/C preform		HfC-ZrC-SiC	CVI; PC; SAPS	oxyacetylene torch	0.017		[192]
	C/ZrC-SiC-HfB_2_	short carbon fiber		ZrC-SiC-HfB_2_	pressureless sintering	oxyacetylene flame	2.46	3.51 × 10^−3^	[193]
Si-Zr-Mo	C/C-ZrB_2_-MoSi_2_	C/C preform		ZrB_2_-MoSi_2_	plasma spraying	oxypropylene flame	1.91	4.8 × 10^−4^	[194]
	C/C-SiC-ZrB_2_/MoSi_2_	2.5D SiC-coated C/C preform		SiC-ZrB_2_/MoSi_2_	SAPS;	oxyacetylene torch	0.44	1.67 × 10^−3^	[195]
	C/C-Mo-ZrB_2_-MoSi_2_-SiC	2D C/SiC preform	PyC	SiC-ZrB_2_-MoSi_2_-SiC/Mo	HVOF; CVI; SAPS	CO_2_ laser beam			[196]
	C/SiOC-MoSi_2_-SiO_2_-SiC/ZrB_2_-MoSi_2_-SiC	carbon fiber needled felt		MoSi_2_-SiO_2_-SiC/ZrB_2_-MoSi_2_-SiC	PIP; slurry and precursor infiltration	oxyacetylene torch			[197]
Si-Ta-Hf	C/HfC-TaC/HfC-SiC	2D needled C/C preform		HfC-TaC/HfC-SiC	SAPS	oxyacetylene torch			[198]
	C/C-Hf-Ta-Si	2.5D C/C preform		Hf-Ta-Si-C	CVD;	oxyacetylene torch	0.03	1.17 × 10^−4^	[199]
	C/C-SiC-HfC-TaC	2D SiC-coated C/C preform		HfC-TaC	PC; SAPS	oxyacetylene torch	0.35	1.05 × 10^−3^	[200]
Si-Zr-Ti	C/C-ZrC-TiC-SiC	2.5D needled C/C preform	PyC	ZrC-TiC-SiC	reactive infiltration	oxyacetylene torch	2.6	8.2 × 10^−4^	[201]
	C/C-SiC-ZrC-TiC	needled C/C fabrics	PyC	SiC-ZrC-TiC	RMI; CVI	oxyacetylene torch	0.008		[202]
	C/C-ZrC-SiC/TiC	2.5D needled C/C preform		ZrC-SiC/TiC	SAPS; SSP; CVI;	oxyacetylene flames		1 × 10^−3^	[203]
Si-Ti	C/SiC-Ti_3_SiC_2_	carbon cloths	PyC	SiC-Ti_3_SiC_2_	LSI; CVI; SI	oxyacetylene torch	6.3	0.024	[204]
	C/C-SiC-Ti_3_SiC_2_	C/TiC preform		SiC-Ti_3_SiC_2_	LSI	oxyacetylene flame	11.8	0.06	[205]
Si-Y	C/C-SiC-Y_2_SiO_5_	2D needle carbon fabric		SiC-Y_2_SiO_5_	TCVI; PC; SPS	oxyacetylene torch	0.031	2.6 × 10^−3^	[206]
	C/C-Y_2_SiO_5_-SiC	2D C/C preform		Y_2_SiO_5_-SiC	PC; SPS	oxyacetylene torch	0.035	3.43 × 10^−3^	[207]
Si-Zr-Cr	C/C-ZrB_2_-CrSi_2_-SiC-Si	2D C/C preform		ZrB_2_-CrSi_2_-SiC-Si/SiC	PC	corundum tube furnace			[208]
	C/C-SiC-Cr-ZrC	2D C/C preform		SiC-Cr-ZrC	TCVI; SAPS	oxyacetylene flame			[209]
Si-Hf-Ti	C/C-HfC-TiC-SiC	C/C		HfC, TiC and SiC coating	VPS; CVR	ICP plasma wind tunnel			[172]
Si-Ti-Ta	C/C-SiC-TiC-TaC	2/2 C/C twill carbon cloth		SiC-TiC-TaC	MI; SPS;	oxyacetylene flame	3.9	0.0022	[210]
Zr-Hf	C/C-HfC-ZrC	C/C preform		HfC-ZrC	CVD;	oxyacetylene torch			[211]
Hf-Ta-Zr	C/HfC-TaC(HfC-ZrC)	C/C preform		HfC-TaC/HfC-ZrC	CVD;	oxyacetylene torch	0.63	2.1 × 10^−4^	[212]
SiZrHfTiCr	C/C-(HfZrTiCr)B_2_-SiC-Si	C/C preform		(Hf_1/4_Zr_1/4_Ti_1/4_Cr_1/4_)B_2_-SiC-Si	SP; GRSI	oxyacetylene ablator	0.37	1.5 × 10^−4^	[29]
SiZrAlCr	C/C-ZrC-SiC-Al_2_O_3_-Cr	C/C-ZrC-SiC preform		Al_2_O_3_-SiC-ZrC-Cr	RMI; SI; plasma spray	oxyacetylene torch	0.52	4.7 × 10^−4^	[213]
Si-Zr-V	C/C-ZrC-SiC-V_0_._9_-Si_0_._1_	3D needled carbon preform		ZrC-SiC-V_0_._9_-Si_0_._1_	RMI;	oxyacetylene torch	0.25	4.3 × 10^−4^	[214]
Si-Mo/Ti	C/C-(Mo,Ti)Si_2_-SiC	porous C/C preform		(Mo,Ti)Si_2_-SiC	RMI	oxyacetylene torch	0.01	2 × 10^−3^	[215]
Si-Mo	C/C-SiC-MoSi_2_	porous C/C preform		SiC-MoSi_2_	VFI	oxyacetylene torch	1.34	3.5 × 10^−3^	[216]
SiZrMoTa	C/SiCO-TaSi_2_-MoSi_2_-ZrO_2_	carbon felts		TaSi_2_-MoSi_2_-ZrO_2_	sol-gel; pyrolysis	oxyacetylene flame	0.4	8.33 × 10^−4^	[217]
SiZrCrY	C/C-ZrSi_2_-CrSi_2_-Y_2_O_3_/SiC	2D SiC-coated C/C preform		ZrSi_2_-CrSi_2_-Y_2_O_3_/SiC	SAPS;	oxyacetylene torch	0.16	1 × 10^−3^	[218]
SiZrCrAl	C/C-ZrC-SiC-Al-Cr	2.5D needled C/C preform		ZrC-SiC-Al-Cr	CVD; RMI	oxyacetylene torch	0.02	2.5 × 10^−4^	[219]
Si-Zr-La/Y	C/SiC-ZrC-La_2_O_3_; C/SiC-ZrC-Y_2_O_3_	3D needled felt		SiC-ZrC-La_2_O_3_; SiC-ZrC-Y_2_O_3_	CVI; RMI; PIP	oxyacetylene torch	1.19; 4.52	9.93 × 10^−3^; 0.0178	[220]
Si-Mo-(Ti/Al)	C/C-MoSi_2_-SiC-(Ti/Al)	needle-punched C/C preform	PyC	MoSi_2_-SiC-(Ti/Al)	CVI	oxyacetylene torch	0.01	2 × 10^−3^	[221]
Si-Mo-Hf-W	C/ZrB_2_-SiC-MoSi_2_; C/ZrB_2_-SiC-HfSi_2_; C/ZrB_2_-SiC-WSi_2_;	short carbon fiber		ZrB_2_-SiC-MoSi_2_; ZrB_2_-SiC-HfSi_2_; ZrB_2_-SiC-WSi_2_;	ball-milling; hot-pressing	oxyacetylene torch			[222]
Si-Zr-Y	C/C-ZrB_2_-SiC-Y_2_O_3_/SiC	C/C preform		ZrB_2_-SiC-Y_2_O_3_/SiC	PC; APS	muffle furnace			[27]
Si-Zr-Sm	C/C-ZrB_2_/SiC-Sm_2_O_3_	C/C preform		ZrB_2_/SiC-Sm_2_O_3_	APS; IPS	plasma torch	0.319		[223]
Si-Cu	C/C-SiCW-Cu	carbon fiber bundle		SiCW-Cu	CVD; CVI;	oxyacetylene torch	4.56	8 × 10^−3^	[224]
Si-Nd	C/C-Si-SiC-SiO_2_-Nd_2_O_3_	SiC coated C/C preform		Si-SiC-SiO_2_-Nd_2_O_3_	CVI; laser cladding	laser-ablation			[225]
Si-Al	C/C-Al20Si/graphite	3D needled C/C preform		Al20Si/graphite	GCVI;	combustion chamber			[226]
Si-Zr-Ta	C/SiC-ZrB_2_-Ta_x_C_y_	carbon fiber cloth mat		SiC-ZrB_2_-Ta_x_C_y_	RHP; PIP	oxyacetylene torch	1.33	1.9 × 10^−4^	[227]
Si-Zr-Nb	C/SiC-NbC-ZrC	2D C/C preform		SiC-NbC-ZrC	SAPS	oxyacetylene torch	0.48	1.3 × 10^−4^	[228]
Si-La	C/C-SiC-La_2_O_3_	2.5D carbon fiber felts	PyC	SiC-La_2_O_3_	PIP; CVI;	plasma generator	0.722	0.0333	[229]
SiTiZrHfNbTa	C/(TiZrHfNbTa)C-SiC	3D-needled carbon fiber	PyC/SiC	(TiZrHfNbTa)C-SiC	PIP; CVI;	air plasma torch	2.60	2.89 × 10^−3^	[230]
Si-Zr-V	C/C-ZrC-SiC-V	C/C preform		ZrC-SiC-V	RIM	oxyacetylene torch	2	7 × 10^−4^	[231]
Si-Zr-Cu	C/C-SiC-ZrC-Cu	needled carbon fiber felts	PyC	SiC-ZrC-Cu	CVI; PIP; VPI	oxyacetylene flame	3.4	3.5 × 10^−3^	[232]

Note *: MR refers to mass ablation rate; LR is liner ablation rate.

## Data Availability

Data sharing not applicable.

## Sample Availability

Not available.

## References

[1] Wu J., Wang H., Wang C., Zhang Z., Tang Y., Hou Z., Wan S., Wu D., Tan Z., Ouyang X. (2022). High-Pressure synthesis of Al_2_O_3_-cBN-hBN Self-lubricating ceramic. Mater. Des..

[2] Liu R., Yang L., Miao H., Jiang M., Wang Y., Liu X., Wan F. (2022). Influence of the SiC matrix introduction time on the microstructure and mechanical properties of C_f_/Hf_0.5_Zr_0.5_C-SiC ultra-high temperature composites. Ceram. Int..

[3] Gao H., Luo F., Wen Q., Duan S., Zhou W., Zhu D. (2018). Influence of different matrices on the mechanical and microwave absorption properties of SiC fiber-reinforced oxide matrix composites. Ceram. Int..

[4] Glass D.E., Splinter S.C. Active oxidation of a uhtc-based cmc. Proceedings of the International Astronautical Congress.

[5] Galizia P., Sciti D. (2023). Disclosing residual thermal stresses in UHT fibre-reinforced ceramic composites and their effect on mechanical behaviour and damage evolution. Compos. Part B Eng..

[6] Nisar A., Ariharan S., Venkateswaran T., Sreenivas N., Balani K. (2017). Effect of carbon nanotube on processing, microstructural, mechanical and ablation behavior of ZrB_2_-20SiC based ultra-high temperature ceramic composites. Carbon.

[7] Li Z., Xiao P., Xiong X. (2007). Research progress of continuous fiber reinforced ceramic matrix composites. Powder Metall. Mater. Sci. Eng..

[8] Bajpai S., Bhadauria A., Venkateswaran T., Singh S.S., Balani K. (2022). Spark plasma joining of HfB_2_-ZrB_2_ based Ultra High Temperature Ceramics using Ni interlayer. Mater. Sci. Eng. A.

[9] Ren J., Duan Y., Lv C., Luo J., Zhang Y., Fu Y. (2021). Effects of HfC/PyC core-shell structure nanowires on the microstructure and mechanical properties of Hf_1_-xZrxC coating. Ceram. Int..

[10] Vinci A., Silvestroni L., Gilli N., Zoli L., Sciti D. (2022). Advancements in carbon fibre reinforced ultra-refractory ceramic composites: Effect of rare earth oxides addition. Compos. Part A Appl. Sci. Manuf..

[11] Xu B., An Y., Wang P., Jin X., Zhao G. (2017). Microstructure and ablation behavior of double anti-oxidation protection for carbon-bonded carbon fiber composites. Ceram. Int..

[12] Du B., He C., Qian J., Wang X., Cai M., Shui A. (2019). Ablation behaviors and mechanism of ultra-thick anti-oxidation layer coating on carbon-bonded carbon fiber composites. J. Am. Ceram. Soc..

[13] Cheng Y., Lyu Y., Xie Y., Zhang C., Feng J., Xun L., Zhang W., Han W., Zhou S., Hu P. (2022). Starting from essence to reveal the ablation behavior and mechanism of 3D PyC C_f_/ZrC-SiC composite. Corros. Sci..

[14] Inoue R., Arai Y., Kubota Y., Goto K., Kogo Y. (2018). Oxidation behavior of carbon fiber-dispersed ZrB_2_-SiC-ZrC triple phase matrix composites in an oxyhydrogen torch environment. Ceram. Int..

[15] Kubota Y., Arai Y., Yano M., Inoue R., Goto K., Kogo Y. (2019). Oxidation and recession of plain weave carbon fiber reinforced ZrB_2_-SiC-ZrC in oxygen–hydrogen torch environment. J. Eur. Ceram. Soc..

[16] Vinci A., Reimer T., Zoli L., Sciti D. (2021). Influence of pressure on the oxidation resistance of carbon fiber reinforced ZrB_2_/SiC composites at 2000 and 2200 °C. Corros. Sci..

[17] Ogasawara T., Aoki T., Hassan M.S.A., Mizokami Y., Watanabe N. (2011). Ablation behavior of SiC fiber/carbon matrix composites under simulated atmospheric reentry conditions. Compos. Part A Appl. Sci. Manuf..

[18] Fang G., Gao X., Song Y. (2023). A Review on ceramic matrix composites and environmental barrier coatings for aero-engine: Material development and failure analysis. Coatings.

[19] Tejero-Martin D., Bennett C., Hussain T. (2021). A review on environmental barrier coatings: History, current state of the art and future developments. J. Eur. Ceram. Soc..

[20] Xiang Y., Li W., Wang S., Chen Z.-H. (2012). Oxidation behavior of oxidation protective coatings for PIP–C/SiC composites at 1500 °C. Ceram. Int..

[21] Yang X., Huang Q., Su Z., Chang X., Chai L., Liu C., Xue L., Huang D. (2013). Resistance to oxidation and ablation of SiC coating on graphite prepared by chemical vapor reaction. Corros. Sci..

[22] Fang X., Liu F., Lu B., Feng X., Kleebe H.J. (2015). Bio-Inspired Microstructure Design to Improve Thermal Ablation and Oxidation Resistance: Experiment on SiC. J. Am. Ceram. Soc..

[23] Wang Y., Chen Z., Yu S. (2016). Ablation behavior and mechanism analysis of C/SiC composites. J. Mater. Res. Technol..

[24] Binner J., Porter M., Baker B., Zou J., Venkatachalam V., Diaz V.R., D’Angio A., Ramanujam P., Zhang T., Murthy T.S.R.C. (2019). Selection, processing, properties and applications of ultra-high temperature ceramic matrix composites, UHTCMCs—A review. Int. Mater. Rev..

[25] Liu L., Li B., Feng W., Tang C., Zhang J., Yao X., Yang Z., Guo Y., Wang P., Zhang Y. (2021). Effect of loading spectrum with different single pulsing time on the cyclic ablation of C/C-SiC-ZrB_2_-ZrC composites in plasma. Corros. Sci..

[26] Ren X.-R., Wang W.-G., Sun K., Hu Y.-W., Xu L.-H., Feng P.-Z. (2022). Preparation of MoSi_2_-modified HfB_2_-SiC ultra high temperature ceramic anti-oxidation coatings by liquid phase sintering. New Carbon Mater..

[27] Lin H., Liu Y., Liang W., Miao Q., Zhou S., Sun J., Qi Y., Gao X., Song Y., Ogawa K. (2022). Effect of the Y_2_O_3_ amount on the oxidation behavior of ZrB_2_-SiC-based coatings for carbon/carbon composites. J. Eur. Ceram. Soc..

[28] Chen B.-W., Ni D.-W., Lu J., Cai F.-Y., Zou X.-G., Liao C.-J., Wang H.-D., Dong S.-M. (2022). Long-term and cyclic ablation behavior of La_2_O_3_ modified C_f_/ZrB_2_-SiC composites at 2500 °C. Corros. Sci..

[29] Zhang P., Cheng C., Xu M., Liu B., Zhu X., Fu Q. (2022). High-entropy (Hf_0.25_Zr_0.25_Ti_0.25_Cr_0.25_)B_2_ ceramic incorporated SiC-Si composite coating to protect C/C composites against ablation above 2400 K. Ceram. Int..

[30] Jin X., Fan X., Lu C., Wang T. (2018). Advances in oxidation and ablation resistance of high and ultra-high temperature ceramics modified or coated carbon/carbon composites. J. Eur. Ceram. Soc..

[31] Jiang Y., Ni D., Chen B., Lu J., Cai F., Zou X., Liao C., Wang H., Dong S. (2021). Fabrication and optimization of 3D-C_f_/HfC-SiC-based composites via sol-gel processing and reactive melt infiltration. J. Eur. Ceram. Soc..

[32] Padture N.P. (2016). Advanced structural ceramics in aerospace propulsion. Nat. Mater..

[33] Lee S., Park G., Kim J.G., Paik J.G. (2019). Evaluation System for Ablative Material in a High-Temperature Torch. Int. J. Aeronaut. Space Sci..

[34] Fan Y., Yang D., Mei H., Xiao S., Yao Y., Cheng L., Zhang L. (2022). Tuning SiC nanowires interphase to improve the mechanical and electromagnetic wave absorption properties of SiC_f_/SiC_nw_/Si_3_N_4_ composites. J. Alloys Compd..

[35] Mungiguerra S., Silvestroni L., Savino R., Zoli L., Esser B., Lagos M., Sciti D. (2022). Qualification and reusability of long and short fibre-reinforced ultra-refractory composites for aerospace thermal protection systems. Corros. Sci..

[36] Jenkins M.G., Mello M.D. (1996). Fabrication, Processing, and Characterization of Braided, Continuous SiC Fiber-Reinforced/CVI SiC Matrix Ceramic Composites. Mater. Manuf. Process..

[37] Grantham T., Tanner G., Molina R., Duong N.-M., Koo J.H. Ablation, thermal, and morphological properties of sic fibers reinforced ceramic matrix composites. Proceedings of the 56th AIAA/ASCE/AHS/ASC Structures, Structural Dynamics, and Materials Conference.

[38] Carney C.M. (2018). 5.10 Ultra-High Temperature Ceramic-Based Composites. Comprehensive Composite Materials II.

[39] Lee Y.-J., Joo H.J. (2004). Ablation characteristics of carbon fiber reinforced carbon (CFRC) composites in the presence of silicon carbide (SiC) coating. Surf. Coat. Technol..

[40] Gao P., Xiao H., Wang H., Jin Z. (2005). A study on the oxidation kinetics and mechanism of three-dimensional (3D) carbon fiber braid coated by gradient SiC. Mater. Chem. Phys..

[41] Tang S., Deng J., Liu W., Yang K. (2006). Mechanical and ablation properties of 2D-carbon/carbon composites pre-infiltrated with a SiC filler. Carbon.

[42] Chen Z., Fang D., Miao Y., Yan B. (2008). Comparison of morphology and microstructure of ablation centre of C/SiC composites by oxy-acetylene torch at 2900 and 3550 °C. Corros. Sci..

[43] Chen Z., Yan B. (2009). Morphology and Microstructure of Three-Dimensional Orthogonal C/SiC Composites Ablated by an Oxyacetylene Flame at 2900 °C. Int. J. Appl. Ceram. Technol..

[44] Li S., Feng Z., Liu Y., Yang W., Zhang W., Cheng L., Zhang L. (2010). Microstructural evolution of coating-modified 3D C/SiC composites after annealing in wet oxygen at different temperatures. Corros. Sci..

[45] Nie J., Xu Y., Zhang L., Fan S., Xu F., Cheng L., Ma J., Yin X. (2010). Microstructure, Thermophysical, and Ablative Performances of a 3D Needled C/C-SiC Composite. Int. J. Appl. Ceram. Technol..

[46] Wei L., Yang X., Song W., Yan M., Zhao-hui C. (2013). Ablation behavior of three-dimensional braided C/SiC composites by oxyacetylene torch under different environments. Ceram. Int..

[47] Safi S., Kazemzadeh A. (2013). MCMB–SiC composites; new class high-temperature structural materials for aerospace applications. Ceram. Int..

[48] Liu L., Li H., Feng W., Shi X., Wu H., Zhu J. (2013). Effect of surface ablation products on the ablation resistance of C/C–SiC composites under oxyacetylene torch. Corros. Sci..

[49] Liu L., Li H., Shi X., Feng W., Wang Y., Yao D. (2013). Effects of SiC addition on the ablation properties of C/C composites in different heat fluxes under oxyacetylene torch. Vacuum.

[50] Ni C., Li K., Liu L., Li H., Fu Q., Guo L., Liu N. (2014). Ablation mechanism of SiC coated C/C composites at 0° angle in two flame conditions under an oxyacetylene flame. Corros. Sci..

[51] Cui Y., Li A., Li B., Ma X., Bai R., Zhang W., Ren M., Sun J. (2014). Microstructure and ablation mechanism of C/C–SiC composites. J. Eur. Ceram. Soc..

[52] Luo L., Wang Y., Liu L., Duan L., Wang G., Lu Y. (2016). Ablation behavior of C/SiC composites in plasma wind tunnel. Carbon.

[53] Kumar S., Kumar A., Mala R.B., Mokhasunavisu R.R. (2015). Fabrication and Ablation Studies of 4D C/SiC Composite Nozzle Under Liquid Propulsion. Int. J. Appl. Ceram. Technol..

[54] Fang X., Liu F., Xia B., Ou D., Feng X. (2016). Formation mechanisms of characteristic structures on the surface of C/SiC composites subjected to thermal ablation. J. Eur. Ceram. Soc..

[55] Jiao X., Li T., Li Y., Zhang Z., Dai F., Feng Z. (2017). Oxidation behavior of SiC/glaze-precursor coating on carbon/carbon composites. Ceram. Int..

[56] Jin X., Fan X., Jiang P., Wang Q. (2017). Microstructure Evolution and Ablation Mechanism of C/C and C/C-SiC Composites Under a Hypersonic Flowing Propane Torch. Adv. Eng. Mater..

[57] Chen L., Yang X., Su Z.A., Fang C., Zeng G., Huang Q. (2018). Fabrication and performance of micro-diamond modified C/SiC composites via precursor impregnation and pyrolysis process. Ceram. Int..

[58] Frueh S.J., Coons T.P., Reutenauer J.W., Gottlieb R., Kmetz M.A., Suib S.L. (2018). Carbon fiber reinforced ceramic matrix composites with an oxidation resistant boron nitride interface coating. Ceram. Int..

[59] Jin W., Si Z., Lu Y., Bei-zhi S., Yi W., Guang-de L., Zhong-fang X., Jie C., Heng-ping H., Yang X. (2018). Oxidation behavior and high-temperature flexural property of CVD-SiC-coated PIP-C/SiC composites. Ceram. Int..

[60] da Silva R.J., Reis R.I., Pardini L.C., Sias D.F., Filho G.P. (2019). Low-Energy Ablation and Low Thermal Diffusivity of a CFRC Composite Modified by SiC. Int. J. Thermophys..

[61] Fan X., Dang X., Ma Y., Yin X., Zhang L., Cheng L. (2019). Microstructure, mechanical and ablation behaviour of C/SiC–Si with different preforms. Ceram. Int..

[62] Tang Y., Yue M., Fang X., Feng X. (2019). Evolution of surface droplets and flow patterns on C/SiC during thermal ablation. J. Eur. Ceram. Soc..

[63] Wang H., Li H.-J., Liu X.-S., Li N., Song Q. (2019). Effects of SiC nanowire decorated with carbon nanosheet on mechanical, heat-dissipation and anti-ablation properties of carbon/carbon composites. Ceram. Int..

[64] Kim J., Kim S., Song S.H., Lee D. (2020). Effects of residual Si on the ablation properties of biomorphic C/SiC composites for reusable thermal protection systems. Adv. Compos. Mater..

[65] Huang D., Tan R., Liu L., Ye C., Zhu S., Fan Z., Zhang P., Wu H., Han F., Liu H. (2021). Preparation and properties of the three-dimensional highly thermal conductive carbon/carbon-silicon carbide composite using the mesophase-pitch-based carbon fibers and pyrocarbon as thermal diffusion channels. J. Eur. Ceram. Soc..

[66] Liu C., Zhang X., Wang G., Wang Z., Gao L. (2021). New ablation evolution behaviors in micro-hole drilling of 2.5D C_f_/SiC composites with millisecond laser. Ceram. Int..

[67] Shi Y.-A., Zha B.-L., Su Q.-D., Wang J.-J., Li S.-Y. (2021). Thermal performance and ablation characteristics of C/C-SiC for thermal protection of hypersonic vehicle. J. Eur. Ceram. Soc..

[68] Xu M., Guo L., Huang J., Zhang P. (2021). Microstructure and resistance to thermal shock of SiC coatings that are prepared on C/C-composite pyrocarbon matrices of various textures. Ceram. Int..

[69] Zhang Y., Liu Y., Cao L., Chen J., Qiu G., Wang J. (2021). Preparation and analysis of micro-holes in C/SiC composites and ablation with a continuous wave laser. J. Eur. Ceram. Soc..

[70] Zhang Z., Fang C., Chen L., Yang X., Shi A., Huang Q., Hu H. (2021). Fabrication, microstructure and ablation resistance of C/C–SiC composites, by using a novel precursor of SiC. Ceram. Int..

[71] Zhao X., Cao Y., Duan L., Li Z., Wang Y. (2021). Low-surface-temperature jump behavior of C/SiC composites prepared via precursor impregnation and pyrolysis in high-enthalpy plasma flows. J. Eur. Ceram. Soc..

[72] Zhu Y., Wei H., Yan L., Cui H. (2021). Morphology and anti-ablation properties of PIP C_f_/C–SiC composites with different CVI carbon content under 4.2 MW/m2 heat flux oxy-acetylene test. Ceram. Int..

[73] Liu N., Guo L., Kou G., Li Y., Yin X. (2022). Ablation behavior of thin-blade co-deposited C/Cx-SiCy composites under the influence of the complex fluid conditions of the oxyacetylene torch. Ceram. Int..

[74] Liu N., Guo L., Kou G., Li Y., Yin X. (2022). The influence of heat treatment on the ablation behavior of the C/Cx-SiCy composites tested by thin-blade under oxyacetylene torch. Ceram. Int..

[75] Xu M., Guo L., Zhang P., Li W. (2022). Effect of pyrolytic carbon texture on ablation behavior of carbon/carbon composites coated with SiC by pack cementation. Ceram. Int..

[76] Guo W., Ye Y., Bai S., Zhu L.A., Li S. (2019). Gel reactive melt infiltration: A new method for large-sized complex-shaped C/C components ceramic modification. Ceram. Int..

[77] Chen Z.F., Wan S.C., Fang D., Zhu J.X., Zhang J.Z. (2013). Morphology and ablation mechanism of C_f_/Si_3_N_4_ composites ablated by oxyacetylene torch at 2200 °C. Corros. Eng. Sci. Technol..

[78] Ding Q., Chen B., Ni D., Ni N., He P., Gao L., Wang H., Zhou H., Dong S. (2021). Improved ablation resistance of 3D-C_f_/SiBCN composites with (PyC/SiC)_3_ multi-layers as interphase. J. Eur. Ceram. Soc..

[79] Yan C., Liu R., Cao Y., Zhang C., Zhang D. (2014). Ablation behavior and mechanism of C/ZrC, C/ZrC–SiC and C/SiC composites fabricated by polymer infiltration and pyrolysis process. Corros. Sci..

[80] Wang J., Duan X., Yang Z., Jia D., Zhou Y. (2014). Ablation mechanism and properties of SiC_f_/SiBCN ceramic composites under an oxyacetylene torch environment. Corros. Sci..

[81] Yang X., Su Z., Huang Q., Chang X., Fang C., Chen L., Zeng G. (2016). Effects of oxidizing species on ablation behavior of C/C-ZrB_2_-ZrC-SiC composites prepared by precursor infiltration and pyrolysis. Ceram. Int..

[82] Zhu Q., Wang G., Liao X., Liang B., Li D., Yang Z., Jia D., Zhou Y., Zhang T., Gao C. (2021). Ablation properties and mechanisms of BN-coated C_f_-reinforced SiBCNZr ceramic composites under an oxyacetylene combustion torch. Ceram. Int..

[83] Tong Y., Zhang H., Hu Y., Zhang P., Hua M., Liang X., Chen Y., Zhang Z. (2021). RMI-C/C-SiC-ZrSi_2_ composite serving in inert atmosphere up to 2100 °C: Thermal shock resistance, microstructure and damage mechanism. Ceram. Int..

[84] Wang Y., Liu W., Cheng L., Zhang L. (2009). Preparation and properties of 2D C/ZrB_2_-SiC ultra high temperature ceramic composites. Mater. Sci. Eng. A.

[85] Tang S., Deng J., Wang S., Liu W. (2007). Fabrication and Characterization of an Ultra-High-Temperature Carbon Fiber-Reinforced ZrB_2_-SiC Matrix Composite. J. Am. Ceram. Soc..

[86] Tang S., Deng J., Wang S., Liu W. (2009). Comparison of thermal and ablation behaviors of C/SiC composites and C/ZrB_2_–SiC composites. Corros. Sci..

[87] Yao X., Li H., Zhang Y., Li K., Fu Q., Peng H. (2013). Ablation Behavior of ZrB_2_-Based Coating Prepared by Supersonic Plasma Spraying for SiC-Coated C/C Composites Under Oxyacetylene Torch. J. Therm. Spray Technol..

[88] Zou X., Fu Q., Liu L., Li H., Wang Y., Yao X., He Z. (2013). ZrB_2_–SiC coating to protect carbon/carbon composites against ablation. Surf. Coat. Technol..

[89] Li H.-J., Yao X.-Y., Zhang Y.-L., Li K.-Z., Guo L.-J., Liu L. (2013). Effect of heat flux on ablation behaviour and mechanism of C/C–ZrB_2_–SiC composite under oxyacetylene torch flame. Corros. Sci..

[90] Zhang Y., Hu Z., Li H., Ren J. (2014). Ablation resistance of ZrB_2_–SiC coating prepared by supersonic atmosphere plasma spraying for SiC-coated carbon/carbon composites. Ceram. Int..

[91] Huang D., Zhang M.-Y., Huang Q.-Z., Wang L.-P., Tang X., Yang X., Tong K. (2015). Fabrication and ablation property of carbon/carbon composites with novel SiC–ZrB_2_ coating. Trans. Nonferrous Met. Soc. China.

[92] Liu L., Li H., Zhang Y., Shi X., Fu Q., Feng W., Feng T. (2015). Effect of ZrB_2_ and SiC distributions on the ablation of modified carbon/carbon composites. Ceram. Int..

[93] Wang D., Zeng Y., Xiong X., Li G., Chen Z., Sun W., Wang Y. (2014). Preparation and ablation properties of ZrB_2_–SiC protective laminae for carbon/carbon composites. Ceram. Int..

[94] Fang X., Liu F., Su H., Liu B., Feng X. (2014). Ablation of C/SiC, C/SiC–ZrO_2_ and C/SiC–ZrB_2_ composites in dry air and air mixed with water vapor. Ceram. Int..

[95] Yang X., Wei L., Song W., Bi-feng Z., Zhao-hui C. (2013). ZrB_2_/SiC as a protective coating for C/SiC composites: Effect of high temperature oxidation on mechanical properties and anti-ablation property. Compos. Part B Eng..

[96] Zhang Y., Hu Z., Yang B., Ren J., Li H. (2015). Effect of pre-oxidation on the ablation resistance of ZrB_2_–SiC coating for SiC-coated carbon/carbon composites. Ceram. Int..

[97] Feng T., Li H.-J., Shi X.-H., Yang X., Wang S.-L. (2013). Oxidation and ablation resistance of ZrB_2_–SiC–Si/B-modified SiC coating for carbon/carbon composites. Corros. Sci..

[98] Liu Y., Fu Q., Wang B., Liu T., Sun J. (2017). The ablation behavior and mechanical property of C/C-SiC-ZrB_2_ composites fabricated by reactive melt infiltration. Ceram. Int..

[99] Ghelich R., Mehdinavaz Aghdam R., Jahannama M.R. (2017). Elevated temperature resistance of SiC-carbon/phenolic nanocomposites reinforced with zirconium diboride nanofibers. J. Compos. Mater..

[100] Liégaut C., Bertrand P., Maillé L., Rebillat F. (2022). UHTC-based matrix as protection for C_f_/C composites: Original manufacturing, microstructural characterisation and oxidation behaviour at temperature above 2000 °C. J. Eur. Ceram. Soc..

[101] Bai Y., Wang Q., Ma Z., Liu Y., Chen H., Ma K., Sun S. (2020). Characterization and ablation resistance of ZrB_2_-xSiC gradient coatings deposited with HPPS. Ceram. Int..

[102] Zhu Y., Cheng L., Ma B., Liu Y., Zhang L. (2018). Effect of CVD ZrB_2_ coating thickness on anti-ablation performance of C/SiC composites. Ceram. Int..

[103] Sciti D., Zoli L., Vinci A., Silvestroni L., Mungiguerra S., Galizia P. (2021). Effect of PAN-based and pitch-based carbon fibres on microstructure and properties of continuous C_f_/ZrB_2_-SiC UHTCMCs. J. Eur. Ceram. Soc..

[104] Kong J.H., Lee S.Y., Son Y.I., Kim D.K. (2023). Synergistic reinforcement effects of ZrB_2_ on the ultra-high temperature stability of C_f_/SiC composite fabricated by liquid silicon infiltration. J. Eur. Ceram. Soc..

[105] Ma Y., Li Q., Dong S., Wang Z., Shi G., Zhou H., Wang Z., He P. (2014). Microstructures and ablation properties of 3D 4-directional C_f_/ZrC–SiC composite in a plasma wind tunnel environment. Ceram. Int..

[106] Wang D., Dong S., Zhou H., Kan Y., Wang Z., Zhu G., Chen X., Cao Y. (2016). Effect of pyrolytic carbon interface on the properties of 3D C/ZrC–SiC composites fabricated by reactive melt infiltration. Ceram. Int..

[107] Li Z., Li H., Zhang S., Wang J., Li W., Sun F. (2012). Effect of reaction melt infiltration temperature on the ablation properties of 2D C/C–SiC–ZrC composites. Corros. Sci..

[108] Li Z., Li H., Zhang S., Li W., Wang J. (2013). Microstructures and ablation properties of C/C−SiC−ZrC composites prepared using C/C skeletons with various densities. Ceram. Int..

[109] Li K.-Z., Jing X., Qian-gang F., He-jun L., Ling-jun G. (2013). Effects of porous C/C density on the densification behavior and ablation property of C/C–ZrC–SiC composites. Carbon.

[110] Xie J., Li K., Li H., Fu Q., Liu L. (2014). Cyclic ablation behavior of C/C–ZrC–SiC composites under oxyacetylene torch. Ceram. Int..

[111] Feng B., Li H., Zhang Y., Liu L., Yan M. (2014). Effect of SiC/ZrC ratio on the mechanical and ablation properties of C/C–SiC–ZrC composites. Corros. Sci..

[112] Zhuang L., Fu Q.-G., Tan B.-Y., Guo Y.-A., Ren Q.-W., Li H.-J., Li B., Zhang J.-P. (2016). Ablation behaviour of C/C and C/C–ZrC–SiC composites with cone-shaped holes under an oxyacetylene flame. Corros. Sci..

[113] Chang Y., Sun W., Xiong X., Chen Z., Wang Y., Hao Z., Xu Y. (2016). Microstructure and ablation behaviors of a novel gradient C/C-ZrC-SiC composite fabricated by an improved reactive melt infiltration. Ceram. Int..

[114] Li J., Yang X., Su Z.-A., Xue L., Zhong P., Li S.-P., Huang Q.-Z., Liu H.-W. (2016). Effect of ZrC–SiC content on microstructure and ablation properties of C/C composites. Trans. Nonferrous Met. Soc. China.

[115] Tong Y., Ye Y., Bai S., Zhang H. (2014). Effects of zirconium addition on microstructure and ablation resistance of carbon fibre reinforced carbon and SiC ceramic matrix composite prepared by reactive melt infiltration. Adv. Appl. Ceram..

[116] Liu C., Cao L., Chen J., Xue L., Tang X., Huang Q. (2013). Microstructure and ablation behavior of SiC coated C/C–SiC–ZrC composites prepared by a hybrid infiltration process. Carbon.

[117] Liu C., Su Z., Huang Q., Chen J., Yang X., Cao L., Yin T., Zhong P. (2014). Ablation behavior of ZrC–SiC coated C/C–ZrC–SiC composites prepared by precursor infiltration pyrolysis combined with reactive melt infiltration. J. Alloys Compd..

[118] Yang X., Wei L., Song W., Chen Z.-H., Zhang Y.-Z. (2015). Ablation behavior and mechanism of SiC/Zr–Si–C multilayer coating for PIP-C/SiC composites under oxyacetylene torch flame. Compos. Part B Eng..

[119] Chen S.A., Zhang C., Zhang Y., Zhao D., Hu H., Zhang Z. (2013). Mechanism of ablation of 3D C/ZrC–SiC composite under an oxyacetylene flame. Corros. Sci..

[120] Xie J., Li K., Li H., Fu Q., Guo L. (2013). Ablation behavior and mechanism of C/C–ZrC–SiC composites under an oxyacetylene torch at 3000 °C. Ceram. Int..

[121] Zhu Y., Hu Y., Cui H., Yan L.-S. (2021). Morphology and anti-ablation properties of composites nozzles under the Φ100 mm H_2_O_2_- polyethylene hybrid rocket motor test. Ceram. Int..

[122] Wang S., Li H., Ren M., Zuo Y., Yang M., Zhang J., Sun J. (2017). Microstructure and ablation mechanism of C/C-ZrC-SiC composites in a plasma flame. Ceram. Int..

[123] Zhou Z., Sun Z., Ge Y., Peng K., Ran L., Yi M. (2018). Microstructure and ablation performance of SiC–ZrC coated C/C composites prepared by reactive melt infiltration. Ceram. Int..

[124] Zhou H., Ni D., He P., Yang J., Hu J., Dong S. (2018). Ablation behavior of C/C-ZrC and C/SiC-ZrC composites fabricated by a joint process of slurry impregnation and chemical vapor infiltration. Ceram. Int..

[125] Zhao Z., Li K., Li W., Liu Q., Kou G., Zhang Y. (2018). Preparation, ablation behavior and mechanism of C/C-ZrC-SiC and C/C-SiC composites. Ceram. Int..

[126] Zhao Z., Li K., Li W., Liu Q., Kou G., Zhang Y. (2018). Ablation behavior of C/C-ZrC-SiC composites prepared by reactive melt infiltration under oxyacetylene torch at two heat fluxes. Ceram. Int..

[127] Zhao Z., Li K., Kou G., Liu T., Li W. (2018). Mechanical properties and ablation behavior of C/C-ZrC and C/C-ZrC-SiC composites prepared by precursor infiltration and pyrolysis combined with chemical vapor infiltration. Ceram. Int..

[128] Zhao R., Hu C., Wang Y., Pang S., Li J., Tang S., Cheng H.-M. (2022). Construction of sandwich-structured C/C-SiC and C/C-SiC-ZrC composites with good mechanical and anti-ablation properties. J. Eur. Ceram. Soc..

[129] Zeng C., Zhang M., He H., Wang X., Tong K., Wang Y., Xu P., Zheng L., Zeng G., Su Z. (2021). The effect of carbon source addition order during sol-gel process on the properties of C/C–ZrC–SiC composites. Ceram. Int..

[130] Xie J., Li K., Sun G., Li H., Su X., Han Y., Li T. (2019). Effects of surface structure unit of 2D needled carbon fiber preform on the microstructure and ablation properties of C/C-ZrC-SiC composites. Ceram. Int..

[131] Wu X., Su Z., Huang Q., Tong K., Xie X., Zeng C. (2020). Effect of ZrC particle distribution on the ablation resistance of C/C-SiC-ZrC composites fabricated using precursor infiltration pyrolysis. Ceram. Int..

[132] Tian X., Shi X., Yang L., Li H., Lin H. (2021). Preparation and ablation properties of SiC nanowire-reinforced ZrC–SiC coating-matrix integrated C/C composites. Ceram. Int..

[133] Guo W., Bai S., Ye Y. (2021). Polymer-metal slurry reactive melt infiltration: A flexible and controllable ceramic modification strategy for irregular C/C components. Ceram. Int..

[134] Tian T., Sun W., Qing X., Xiong X., Zhang H., Chu Y., Chen Z., Zeng Y. (2020). Intelligent cooling structure design of "Z-pins like" silicon rods to enhance the ablation resistance of C/C-ZrC-SiC composites above 2500 °C. J. Eur. Ceram. Soc..

[135] Qing X., Sun W., Tian T., Xiong X., Zhang H., Chen Z., Zeng Y. (2020). Structural characteristics and ablative behavior of C/C–ZrC–SiC composites reinforced with “Z-pins like” Zr–Si–B–C multiphase ceramic rods. Ceram. Int..

[136] Liu R., Liu X., Wang Y., Miao H., Song C., Qi G., Wan F. (2021). Laser ablation behavior and mechanism of C_f_/SiC–ZrC ultra-high temperature ceramic matrix composite prepared by PIP method. Ceram. Int..

[137] Li B., Li H., Hu X., Feng G., Yao X., Wang P. (2020). Effect of the curvature radius of sharp leading edge parts made of a SiC/ZrC-SiC coated C/C composite on their ablation resistance. J. Eur. Ceram. Soc..

[138] He Q., Li H., Yin X., Wang C., Lu J. (2019). Microstructure, mechanical and anti-ablation properties of SiCnw/PyC core-shell networks reinforced C/C–ZrC–SiC composites fabricated by a multistep method of chemical liquid-vapor deposition. Ceram. Int..

[139] He Q., Li H., Wang C., Zhou H., Lu J. (2019). Microstructure and ablation property of C/C-ZrC-SiC composites fabricated by chemical liquid-vapor deposition combined with precursor infiltration and pyrolysis. Ceram. Int..

[140] He Q., Li H., Wang C., Li T., Lu J. (2019). Microstructure and ablation property of gradient ZrC SiC modified C/C composites prepared by chemical liquid vapor deposition. Ceram. Int..

[141] Feng T., Shi X., Hou W., Tong M., Li H., Lin H., Wen S. (2022). The ablation and mechanical behaviors of C/(SiC-ZrC)_n_ multi-layer structure matrix composites by chemical vapor infiltration. J. Eur. Ceram. Soc..

[142] Feng G., Yu Y., Yao X., Jia Y., Sun J., Li H. (2022). Ablation behavior of single and alternate multilayered ZrC-SiC coatings under oxyacetylene torch. J. Eur. Ceram. Soc..

[143] Feng G., Li H., Yao X., Chen L., Yu Y., Wang H., Chen M. (2021). Ablation behavior of ZrC and ZrO_2_ coatings on SiC coated C/C composites under oxyacetylene torch with different heat fluxes. Ceram. Int..

[144] Chen S.A., Li G., Hu H., Li Y., Mei M. (2017). Microstructure and properties of ablative C/ZrC–SiC composites prepared by reactive melt infiltration of zirconium and vapour silicon infiltration. Ceram. Int..

[145] Chen B.-W., Ni D.-W., Wang J.-X., Jiang Y.-L., Ding Q., Gao L., Zhang X.-Y., Ding Y.-S., Dong S.-M. (2019). Ablation behavior of C_f_/ZrC-SiC-based composites fabricated by an improved reactive melt infiltration. J. Eur. Ceram. Soc..

[146] Li B., Li H., Yao X. (2022). Ablation behaviour of the CVD-(ZrC/SiC)_3_ alternate coating on C/C composites under oxyacetylene torch with different heat fluxes. Ceram. Int..

[147] Feng G., Li H., Yao X., Sun J., Jia Y. (2022). An optimized strategy toward multilayer ablation coating for SiC-coated carbon/carbon composites based on experiment and simulation. J. Eur. Ceram. Soc..

[148] Huang D., Zhang M., Huang Q., Wang L., Xue L., Tang X., He K. (2015). Ablation mechanism of C/C–ZrB_2_–ZrC–SiC composite fabricated by polymer infiltration and pyrolysis with preform of C_f_/ZrB_2_. Corros. Sci..

[149] Zhang Y., Hu H., Zhang P., Hu Z., Li H., Zhang L. (2016). SiC/ZrB_2_–SiC–ZrC multilayer coating for carbon/carbon composites against ablation. Surf. Coat. Technol..

[150] Li H., Liu L., Zhang Y., Li K., Shi X., Zhang Y., Feng W. (2015). Effect of high temperature heat treatment on the ablation of SiC–ZrB_2_–ZrC particles modified C/C composites in two heat fluxes. J. Alloys Compd..

[151] Liu L., Li H., Shi X., Fu Q., Feng W., Yao X., Ni C. (2014). Influence of SiC additive on the ablation behavior of C/C composites modified by ZrB_2_–ZrC particles under oxyacetylene torch. Ceram. Int..

[152] Hu C., Pang S., Tang S., Wang S., Huang H., Cheng H.-M. (2014). Ablation and mechanical behavior of a sandwich-structured composite with an inner layer of C_f_/SiC between two outer layers of C_f_/SiC–ZrB_2_–ZrC. Corros. Sci..

[153] Zhuang L., Fu Q.-G., Liu T.-Y. (2016). Ablation resistance of wedge-shaped C/C-ZrB_2_-ZrC-SiC composites exposed to an oxyacetylene torch. Corros. Sci..

[154] Kannan R., Rangaraj L. (2017). Processing and characterization of C_f_/ZrB_2_-SiC-ZrC composites produced at moderate pressure and temperature. Ceram. Int..

[155] Zhang Y., Wang H., Li T., Fu Y., Ren J. (2018). Ultra-high temperature ceramic coating for carbon/carbon composites against ablation above 2000 K. Ceram. Int..

[156] Tong Y.G., Cai Z.H., Bai S.X., Hu Y.L., Hua M.Y., Xie W., Ye Y.C., Li Y. (2018). Microstructures and properties of Si-Zr alloy based CMCs reinforced by various porous C/C performs. Ceram. Int..

[157] Tong Y., Hu Y., Liang X., Zhang Z., Li Y., Chen Z., Xiong X., Hua M. (2020). Carbon fiber reinforced ZrC based ultra-high temperature ceramic matrix composite subjected to laser ablation: Ablation resistance, microstructure and damage mechanism. Ceram. Int..

[158] Tong Y., Bai S., Hu Y., Liang X., Ye Y., Qin Q.H. (2018). Laser ablation resistance and mechanism of Si-Zr alloyed melt infiltrated C/C-SiC composite. Ceram. Int..

[159] Subha S., Singh D., Venkatanarayanan P.S., Kavitha N. (2018). Experimental investigation of mechanical, thermal, and ablation performance of ZrO_2_/SiC modified C-Ph composites. Int. J. Appl. Ceram. Technol..

[160] Wang P., Li D., Meng J., Wei C., Li S., Geng X., Sun H., Li X., Wu Y., Wen G. (2022). Effect of silicon/graphite ratio and temperature on oxidation protective properties of SiC/ZrB_2_–SiC coatings prepared by pack cementation. Ceram. Int..

[161] Torabi S., Valefi Z., Ehsani N. (2022). Evaluation of oxy-acetylene flame angle effect on the erosion resistance of SiC/ZrB_2_–SiC/ZrB_2_ multilayer coatings fabricated by the shielding shrouded plasma spray technique. Ceram. Int..

[162] Hu P., Cheng Y., Xie M., Yang Y., Liu C., Qu Q., Zhang X., Du S. (2018). Damage mechanism analysis to the carbon fiber and fiber-ceramic interface tailoring of C_f_/ZrC-SiC using PyC coating. Ceram. Int..

[163] Chen X., Feng Q., Kan Y., Ni D., Zhou H., Gao L., Zhang X., Ding Y., Dong S. (2020). Effects of preform pore structure on infiltration kinetics and microstructure evolution of RMI-derived C_f_/ZrC-ZrB_2_-SiC composite. J. Eur. Ceram. Soc..

[164] Patra N., Al Nasiri N., Jayaseelan D.D., Lee W.E. (2018). Thermal properties of C_f_/HfC and C_f_/HfC-SiC composites prepared by precursor infiltration and pyrolysis. J. Eur. Ceram. Soc..

[165] Vinci A., Zoli L., Sciti D., Watts J., Hilmas G.E., Fahrenholtz W.G. (2019). Influence of fibre content on the strength of carbon fibre reinforced HfC/SiC composites up to 2100 °C. J. Eur. Ceram. Soc..

[166] Xu J., Sun W., Xiong X., Xu Y., Deng N., Li W., Chen Z. (2020). Microstructure and ablation behaviour of a strong, dense, and thick interfacial Zr_x_Hf_1−x_C/SiC multiphase bilayer coating prepared by a new simple one-step method. Ceram. Int..

[167] Abdollahi A., Valefi Z., Ehsani N. (2020). Erosion mechanism of ternary-phase SiC/ZrB_2_-MoSi_2_-SiC ultra-high temperature multilayer coating under supersonic flame at 90° angle with speed of 1400 m/s (Mach 4). J. Eur. Ceram. Soc..

[168] Jia J., Li C., Chen Q., Bai S., Chang J., Xiong D., Gao M., Li S., Xiao J. (2022). Effects of SiC content on the mechanical and thermophysical properties of 3D C_f_/SiC–Al composites. Ceram. Int..

[169] Tan W., Li K., Li H., Zhang J., Ni C., Cao A., Ma C. (2015). Ablation behavior and mechanism of C/C-HfC-SiC composites. Vacuum.

[170] Luo L., Wang Y., Duan L., Liu L., Wang G. (2016). Ablation behavior of C/SiC-HfC composites in the plasma wind tunnel. J. Eur. Ceram. Soc..

[171] Wen Q., Luan X., Wang L., Xu X., Ionescu E., Riedel R. (2019). Laser ablation behavior of SiHfC-based ceramics prepared from a single-source precursor: Effects of Hf-incorporation into SiC. J. Eur. Ceram. Soc..

[172] Chinnaraj R.K., Hong S.M., Kim H.S., Oh P.Y., Choi S.M. (2020). Ablation Experiments of Ultra-High-Temperature Ceramic Coating on Carbon–Carbon Composite Using ICP Plasma Wind Tunnel. Int. J. Aeronaut. Space Sci..

[173] Wang P., Li H., Jia Y., Zhang Y., Yuan R. (2017). Ablation resistance of HfB_2_-SiC coating prepared by in-situ reaction method for SiC coated C/C composites. Ceram. Int..

[174] Tong M., Fu Q., Hu D., Zhou L., Feng T. (2021). Improvement of ablation resistance of CVD-HfC/SiC coating on hemisphere shaped C/C composites by introducing diffusion interface. J. Eur. Ceram. Soc..

[175] Duan L., Zhao X., Wang Y. (2017). Comparative ablation behaviors of C/SiC-HfC composites prepared by reactive melt infiltration and precursor infiltration and pyrolysis routes. Ceram. Int..

[176] Tang Z., Yi M., Xiang Q., Du Y., Peng K. (2021). Mechanical and ablation properties of a C/C-HfB_2_-SiC composite prepared by high-solid-loading slurry impregnation combined with precursor infiltration and pyrolysis. J. Eur. Ceram. Soc..

[177] Zhang P., Fu Q., Cheng C., Sun J., Zhang J., Xu M., Zhu X. (2021). Microstructure evolution of in-situ SiC-HfB_2_-Si ternary coating and its corrosion behaviors at ultra-high temperatures. J. Eur. Ceram. Soc..

[178] Zhang J., Zhang Y., Zhang T., Chen R., Zhu X. (2022). Cyclic ablation behavior and microstructure evolution of multi-layer coating on C/C composites under oxyacetylene torch. Ceram. Int..

[179] Luan X., Yuan J., Wang J., Tian M., Cheng L., Ionescu E., Riedel R. (2016). Laser ablation behavior of C_f_/SiHfBCN ceramic matrix composites. J. Eur. Ceram. Soc..

[180] Feng T., Hou W., Tong M., Li H., Lin H., Wen S. (2021). Effect of SiC_nws_ on flexural strength of SiC_f_/HfC-SiC composites after impact and ablation. J. Eur. Ceram. Soc..

[181] Qu J.-L., Fu Q.-G., Zhang J.-P., Zhang P.-F. (2016). Ablation behavior of TaB_2_-SiC coating for carbon/carbon composites under oxyacetylene torch. Vacuum.

[182] Du B., Hong C., Zhang X., Wang A., Sun Y. (2018). Ablation behavior of advanced TaSi_2_-based coating on carbon-bonded carbon fiber composite/ceramic insulation tile in plasma wind tunnel. Ceram. Int..

[183] Liu F., Li H., Gu S., Yao X., Fu Q. (2019). Ablation behavior and thermal protection performance of TaSi_2_ coating for SiC coated carbon/carbon composites. Ceram. Int..

[184] Chen Z.-K., Xiong X., Li G.-D., Wang Y.-L. (2009). Ablation behaviors of carbon/carbon composites with C-SiC-TaC multi-interlayers. Appl. Surf. Sci..

[185] Jia Y., Li H., Li L., Fu Q., Li K. (2016). Effect of Monolithic LaB_6_ on the Ablation Resistance of ZrC/SiC Coating Prepared by Supersonic Plasma Spraying for C/C Composites. J. Mater. Sci. Technol..

[186] Jia Y., Li H., Feng L., Sun J., Li K., Fu Q. (2016). Ablation behavior of rare earth La-modified ZrC coating for SiC-coated carbon/carbon composites under an oxyacetylene torch. Corros. Sci..

[187] Fang C., Huang B., Yang X., He K., Chen L., Shi A., Zhang Z., Huang Q. (2020). Effects of LaB_6_ on the microstructures and ablation properties of 3D C/C-SiC-ZrB_2_-LaB_6_ composites. J. Eur. Ceram. Soc..

[188] Chen M., Yao X., Feng G., Guo Y. (2020). Anti-ablation performance of La_2_O_3_-modified ZrB_2_ coating on SiC-coated carbon/carbon composites. Ceram. Int..

[189] Jia Y., Li H., Fu Q., Sun J., Li L. (2016). Ablation behavior of ZrC-La_2_O_3_ coating for SiC-coated carbon/carbon composites under an oxyacetylene torch. Ceram. Int..

[190] Lu J., Hao K., Liu L., Li H., Li K., Qu J., Yan X. (2016). Ablation resistance of SiC–HfC–ZrC multiphase modified carbon/carbon composites. Corros. Sci..

[191] Chen Y., Sun W., Xiong X., Chang Y., Xu Y., Peng Z., Tian T., Zeng Y. (2019). Microstructure, thermophysical properties, and ablation resistance of C/HfC-ZrC-SiC composites. Ceram. Int..

[192] Yang Y., Li K., Zhao Z., Liu G. (2017). HfC-ZrC-SiC multiphase protective coating for SiC-coated C/C composites prepared by supersonic atmospheric plasma spraying. Ceram. Int..

[193] Shojaie-Bahaabad M., Hasani-Arefi A. (2020). Ablation properties of ZrC-SiC-HfB_2_ ceramic with different amount of carbon fiber under an oxyacetylene flame. Mater. Res. Express.

[194] Liu X., Deng C., Deng C., Liu M., Zhou K. (2018). Mullite-modified ZrB_2_-MoSi_2_ coating for carbon/carbon composites to withstand long term ablation. Ceram. Int..

[195] Hu D., Fu Q., Liu T., Tong M. (2020). Structural design and ablation performance of ZrB_2_/MoSi_2_ laminated coating for SiC coated carbon/carbon composites. J. Eur. Ceram. Soc..

[196] Cheng S., Geng L., Liu X., Wang Y. (2020). Laser ablation behavior and mechanism of C/SiC coated with ZrB_2_–MoSi_2_–SiC/Mo prepared by HVOF. Ceram. Int..

[197] Sun Y., Dong S., Hong C., Zhang X., Han J., Qu Q. (2020). A novel combination of precursor pyrolysis assisted sintering and rapid sintering for construction of multi-composition coatings to improve ablation resistance of SiOC ceramic modified carbon fiber needled felt preform composites. Ceram. Int..

[198] Feng G., Li H., Yao X., Zhou H., Yu Y., Lu J. (2021). Ablation resistance of HfC-TaC/HfC-SiC alternate coating for SiC-coated carbon/carbon composites under cyclic ablation. J. Eur. Ceram. Soc..

[199] Tong M., Chen C., Fu Q., Feng T., Hou W., Zhang J., Sun J., Zhou L. (2022). Exploring Hf-Ta-O precipitation upon ablation of Hf-Ta-Si-C coating on C/C composites. J. Eur. Ceram. Soc..

[200] Feng G., Li H., Yao X., Chen M., Xue Y. (2019). Ablation resistance of TaC-modified HfC coating prepared by supersonic plasma spraying for SiC-coated carbon/carbon composites. Ceram. Int..

[201] Xu Y., Sun W., Miao C., Shen Y., Chen H., Liu Y., Zhang H., Xiong X. (2021). Ablation properties of C/C-UHTCs and their preparation by reactive infiltration of K_2_MeF_6_ (Me = Zr, Ti) molten salt. J. Eur. Ceram. Soc..

[202] Zeng Y., Wang D., Xiong X., Gao S., Chen Z., Sun W., Wang Y. (2020). Ultra-high-temperature ablation behavior of SiC–ZrC–TiC modified carbon/carbon composites fabricated via reactive melt infiltration. J. Eur. Ceram. Soc..

[203] Zhang Y., Sun J., Guo L., Fan K., Riedel R., Fu Q. (2022). Ablation resistant ZrC coating modified by polymer-derived SiC/TiC nanocomposites for ultra-high temperature application. J. Eur. Ceram. Soc..

[204] Fan X., Yin X., Wang L., Cheng L., Zhang L. (2013). Processing, microstructure and ablation behavior of C/SiC–Ti_3_SiC_2_ composites fabricated by liquid silicon infiltration. Corros. Sci..

[205] Yaghobizadeh O., Sedghi A., Baharvandi H.R. (2019). Effect of Ti_3_SiC_2_ on the ablation behavior and mechanism of C_f_-C-SiC-Ti_3_SiC_2_ composites under oxyacetylene torch at 3000 °C. Ceram. Int..

[206] Zhang J.-P., Fu Q.-G., Zhuang L., Li H.-J., Sun C. (2015). Preparation and Ablation Properties of Y_2_SiO_5_ Coating for SiC-Coated C/C Composites by Supersonic Plasma Spraying. J. Therm. Spray Technol..

[207] Zhang J.-P., Fu Q.-G., Li H.-J., Sun G.-D., Sun C., Nan X.-Y., Li S.-F., Liu L. (2014). Ablation behavior of Y_2_SiO_5_/SiC coating for C/C composites under oxyacetylene torch. Corros. Sci..

[208] Li K., Hu M. (2017). Dynamic oxidation resistance and residual mechanical strength of ZrB_2_-CrSi_2_-SiC-Si/SiC coated C/C composites. Ceram. Int..

[209] Huo C., Guo L., Wang C., Zhou L., Wang J., Li K. (2019). Microstructure and high temperature anti-ablation behavior of Cr-modified ZrC coating for SiC-coated carbon/carbon composites. Ceram. Int..

[210] Makurunje P., Monteverde F., Sigalas I. (2017). Self-generating oxidation protective high-temperature glass-ceramic coatings for C_f_/C-SiC-TiC-TaC UHTC matrix composites. J. Eur. Ceram. Soc..

[211] Ren J., Feng E., Zhang Y., Zhang J., Ding D., Li L. (2021). Influences of deposition temperature, gas flow rate and ZrC content on the microstructure and anti-ablation performance of CVD-HfC-ZrC coating. Ceram. Int..

[212] Ren J., Feng E., Zhang Y., Zhang J., Li L. (2020). Microstructure and anti-ablation performance of HfC-TaC and HfC-ZrC coatings synthesized by CVD on C/C composites. Ceram. Int..

[213] Peng Z., Sun W., Xiong X., Xu Y., Chen Y. (2018). A novel Cr-doped Al_2_O_3_-SiC-ZrC composite coating for ablative protection of C/C-ZrC-SiC composites. J. Eur. Ceram. Soc..

[214] Tian T., Sun W., Qing X., Chu Y., Chen H., Liu Y., Xiong X., Zhang H. (2021). Effect of surface structure unit of 3D needled carbon fiber preform on the ablation improvement of “Z-pins like” V_0.9_-Si_0.1_ rod for C/C–ZrC–SiC. Ceram. Int..

[215] Hao Z., Sun W., Xiong X., Chen Z., Wang Y., Chang Y., Xu Y. (2016). Microstructure and ablation properties of a gradient C_f_/C-XSi_2_-SiC (X = Mo,Ti) composite fabricated by reactive melt infiltration. J. Eur. Ceram. Soc..

[216] Bai Z., Cao L., Huang J., OuYang H., Li C., Zhao X., Wang Y., Yan J. (2016). Microstructures and ablation properties of C_f_/C-SiC-MoSi_2_ composites: Influence of the SiC:MoSi_2_ mass ratio. Ceram. Int..

[217] Li X., Feng J., Jiang Y., Lin H., Feng J. (2018). Preparation and properties of TaSi_2_-MoSi_2_-ZrO_2_-borosilicate glass coating on porous SiCO ceramic composites for thermal protection. Ceram. Int..

[218] Liu F., Li H., Zhang W., Yao X., Fu Q., He X. (2021). Effect of CrSi_2_ on the ablation resistance of a ZrSi_2_-Y_2_O_3_/SiC coating prepared by SAPS. Ceram. Int..

[219] Chang Y., Sun W., Xiong X., Chen Z., Wang Y., Hao Z., Xu Y. (2017). A novel design of Al-Cr alloy surface sealing for ablation resistant C/C-ZrC-SiC composite. J. Eur. Ceram. Soc..

[220] Luo L., Liu J., Duan L., Wang Y. (2015). Multiple ablation resistance of La_2_O_3_/Y_2_O_3_-doped C/SiC–ZrC composites. Ceram. Int..

[221] Hao Z., Sun W., Xiong X., Chen Z., Wang Y. (2016). Effects of Ti/Al addition on the microstructures and ablation properties of C_f_/C–MoSi_2_–SiC composites. J. Eur. Ceram. Soc..

[222] Silvestroni L., Vinci A., Failla S., Zoli L., Rubio V., Binner J., Sciti D. (2019). Ablation behaviour of ultra-high temperature ceramic matrix composites: Role of MeSi_2_ addition. J. Eur. Ceram. Soc..

[223] Xu J., Liu Y., Ma Z., Zhu S., Wang Y., Chen H., Ma K. (2021). Infrared radiative performance and anti-ablation behaviour of Sm_2_O_3_ modified ZrB_2_/SiC coatings. Ceram. Int..

[224] Wang P., Wei F., Zhang H., Yin J., Hao Z., Zhou J. (2022). Effect of SiC whiskers on the mechanical and ablation performances of Cu modified C/C composite. Ceram. Int..

[225] Wang H.-H., Kong J.-A., Ge J., Teng L., Shi X.-H., Li H.-J. (2021). Effects of reflectivity on laser-ablation resistance of the laser-cladding repaired Nd_2_O_3_ modified SiO_2_ coatings on C/C composites. J. Eur. Ceram. Soc..

[226] Liu H., Kang P., Yang W., Zhang N., Sun Y., Wu G. (2020). Ablation behavior of Al20Si/graphite composite nozzle-throats in a solid rocket motor environment. Ceram. Int..

[227] Kannan R., Rangaraj L. (2019). Properties of C_f_/SiC-ZrB_2_-Ta_x_C_y_ composite produced by reactive hot pressing and polymer impregnation pyrolysis (RHP/PIP). J. Eur. Ceram. Soc..

[228] He R., Li K., Gu S., Liu Q. (2020). Comparing ablation properties of NbC and NbC-25 mol.% ZrC coating on SiC-coated C/C composites. Ceram. Int..

[229] Fang C., Yang X., He K., Chen L., Zeng G., Shi A., Huang Q., Huang B. (2019). Microstructure and ablation properties of La_2_O_3_ modified C/C-SiC composites prepared via precursor infiltration pyrolysis. J. Eur. Ceram. Soc..

[230] Cai F., Ni D., Chen B., Ye L., Sun Y., Lu J., Zou X., Zhou H., He P., Zhao T. (2021). Fabrication and properties of C_f_/(Ti_0.2_Zr_0.2_Hf_0.2_Nb_0.2_Ta_0.2_)C-SiC high-entropy ceramic matrix composites via precursor infiltration and pyrolysis. J. Eur. Ceram. Soc..

[231] Tian T., Sun W., Xiong X., Xu Y., Chen Y., Zeng Y., Liu F. (2019). Novel “Z-pins like” vanadium rods prepared by solid phase sintering to improve ablation resistance of the C/C-ZrC-SiC composites. J. Eur. Ceram. Soc..

[232] Weng Y., Yang X., Chen F., Zhang X., Shi A., Yan J., Huang Q. (2022). Effect of CVI SiC content on ablation and mechanism of C/C-SiC-ZrC-Cu composites. Ceram. Int..

